# Infection Rates of Fasciola Intermediate Host Snail Species and Their Distribution in Africa: A Systematic Review and Meta-Analysis

**DOI:** 10.3390/tropicalmed8100467

**Published:** 2023-10-06

**Authors:** Mpumelelo Ian Hadebe, Tawanda Manyangadze, Chester Kalinda, Tafadzwa Mindu, Moses John Chimbari

**Affiliations:** 1 Discipline of Public Health and Nursing, College of Health Sciences, University of KwaZulu-Natal, Durban 4000, South Africa; manyangadze.tawanda@gmail.com (T.M.); ckalinda@ughe.org (C.K.); tafadzwamindu@gmail.com (T.M.); chimbari@ukzn.ac.za (M.J.C.); 2 Department of Geosciences, School of Geosciences, Disaster and Development, Faculty of Science and Engineering, Bindura University of Science Education, Bindura P.O. Box 1020, Zimbabwe; 3 Bill and Joyce Cummings Institute of Global Health, University of Global Health Equity (UGHE), P.O. Box 6955, Kigali 20093, Rwanda; 4 Department of Behavioural Science, Medical and Health Sciences, Great Zimbabwe University, Masvingo P.O. Box 1235, Zimbabwe

**Keywords:** intermediate host snails, radix natalensis, galba truncatula, pseudosuccinea columella, infection rate, fasciola hepatica, fasciola gigantica

## Abstract

This systematic review and meta-analysis aimed to collate the infection rates of *Fasciola* spp. in intermediate host snails and their distribution in Africa. The overall infectivity prevalences of *Galba truncatula, Radix natalensis*, and *Pseudosuccinea columella* are 52%, 8%, and 3%, respectively. The intermediate host snails native to Africa (*R. natalensis* and *G. truncatula*) have been examined more than the invasive *P. columella*. The studies included in the review ranged from 1999 to 2022. North Africa has the highest prevalence of *G. truncatula*, with an infection rate of 52%. The review reveals that naturally infected intermediate host snails (*G. truncatula*, *R. natalensis*, and *P. columella*) are found in various regions of Africa. *G. truncatula* accounts for 22% (from three countries) of the studies included in the review and it was only found in the North African region with the highest overall infection rate of 52%. More studies on infection rate and distribution are needed to effectively control and prevent future transmissions.

## 1. Introduction

Worldwide, fascioliasis is an emerging infection. Climate change effects may result in increased rainfall in some regions and decreased freezing temperatures in others, resulting in the emergence of new habitats for Fasciola and its hosts [1]. New irrigation projects and other man-made environmental changes may result in more appropriate habitats being available [2]. Fascioliasis is one of the most neglected zoonotic diseases in Africa despite being a threat to human and animal health. Good knowledge of the life cycles, host specificity, and geographic distribution of the important medical and veterinary parasites transmitted by intermediate host snails is a crucial requirement in the fight against them. Freshwater snails have been of interest to the research world as they are intermediate hosts for several diseases in animals and humans including fascioliasis. Fascioliasis, a trematode infection, thrives in tropical and subtropical countries. According to Dida et al. [3], before 1992, the total number of reported human cases of fascioliasis was considered to be less than 3000. More recent statistics show that between 2.4 and 17 million people are infected, with an additional 91.1 million people at risk of infection [4]. Trematode infections are still one of Africa’s most common and pervasive tropical diseases, especially in areas near freshwater bodies [5,6]. Fascioliasis is transmitted through two different hosts (intermediate and definitive). In nature, freshwater snails serve as the intermediate hosts for the development of its asexual stage. Humans are accidental hosts, but cattle and other ruminants serve as the final hosts for the sexual stage. The spread of the parasites and their vectors to new areas can be facilitated by migration, globalization, and the import and export of livestock. Together, all of these factors may lead to an expansion of endemic regions and a rise in the prevalence of Fasciola in livestock as well as in humans [6,7,8]. Because herding communities are constantly near the cattle they keep, fascioliasis is a disease that is highly prevalent in these communities [9]. Sripa and Piratae [10] suggested that it is possible to check for local transmission using knowledge about a particular type of snail in a given area. Over a million people in 90 countries are estimated to have been infected by parasitic diseases involving snail intermediate hosts [6]. The transmission dynamics of fascioliasis will be further complicated by climate change as it may change the presence, distribution, abundance, and infection rates of the intermediate host snails. Climate change may also result in the changing of interactions between animals and humans with the *fasciola*-infested waterbodies and pastures through changes in livelihoods.

Fascioliasis is caused by infection of trematodes belonging to the genus *Fasciola* spp. These *Fasciola* spp. are zoonotic pathogens whose common causative agents are *Fasciola hepatic*a (Linnaeus, 1758) and *Fasciola gigantica (*Cobbold, 1855; Galba Schrank, 1803). The presence and distribution of intermediate host snails determine the geographic range of fascioliasis [11]. According to Moema and Baker [12] in Africa, *Radix natalensis* (Krauss, 1848) is involved in the transmission of *F. gigantica* whereas *Galba truncatula* (Muller, 1774) is involved in the transmission of *F. hepatica*, with any epidemiological cross-over being regarded to be unusual [13]. *Galba truncatula* is an intermediate host of the liver fluke *F. hepatica*, which is mostly accountable for human disease. It is distinguished by its amphibious lifestyle, adaptation to cooler habitats, and capacity to endure drought periods and other adverse climatic circumstances in unstable waterbodies [14]. It has been established that the invasive *Pseudosuccinea columella* (Say, 1817) serves as an intermediate host for both *F. hepatica* and *F. gigantica* in numerous countries [15]. Mage et al. [16] carried out a study in Central France over a period of 10–12 years to analyze the changes in the prevalence of fascioliasis. The infectivity prevalence of snails ranged from 4.7% (1989) to 7.2% (1993) and slowly decreased after that date reaching 3.3% (in 2000). Furthermore, Cucher et al. [17] obtained two samples from Argentina which were analyzed and identified as *P. columella* and the infection rate was 17.5% and 51.3% by direct examination and PCR, respectively. A study by [18] showed that *P. columella* can coexist with *R. natalensis* in some parts of South Africa. Fascioliasis is unfortunately not a recognized and reportable disease in many of the least developed countries, which are ravaged by poverty and infectious diseases. The first vital components in any planned intervention strategy include generating knowledge about this disease and raising awareness within the affected communities [19]. This systematic meta-analysis aimed to determine the infection rate of *Fasciola* spp. transmitted by intermediate host snails and their distribution in Africa. The results of this study show the role of these intermediate host snail species in fascioliasis transmission and its potential presence in both humans and animals and regional hotspots in different parts of Africa.

## 2. Methods

### 2.1. Search Strategies and Inclusion Criteria

The search was conducted in three search databases, Google Scholar, Pubmed, Scopus, and other sources (reference list of included articles). The search terms used were as follows: “*Fasciola* intermediate host”, “intermediate host snails”, “snail intermediate host”, “intermediate host”, “freshwater snails”, “freshwater snail host”, “snail vector”, “malacology survey”, “*Lymnaea columella*”, “*Lymnaea natalensis*”, “*Lymnaea truncatula*”, “*Pseudosuccinea columella*”, “*Radix natalensis*”, “*Galba truncatula*”, “infection”, “infection rate”, “intensity”, “prevalence”, “incidence”, “Fascioliasis”, “liver fluke”, “*Fasciola hepatica*”, “*Fasciola gigantica*”, “*Fasciola* sp.”, “Africa”. These search terms were combined using the Boolean operators (AND; OR) to construct search phrases. The last phrase “Africa” was further categorized into African regions in the following manner: North African countries—Algeria OR Egypt OR Libya OR Morocco OR Sudan OR Tunisia OR and Western Sahara; Central or Middle African countries—Angola OR Cameroon OR Central African Republic OR Chad OR Congo Republic—Brazzaville OR Democratic Republic of Congo OR Equatorial Guinea OR Gabon OR and São Tomé and Principe; Southern African countries—Botswana OR Lesotho OR Namibia OR South Africa OR Swaziland OR Lesotho; OR Madagascar OR Malawi OR Mauritius OR Mozambique OR Zambia OR and Zimbabwe; East African countries—Burundi OR Comoros OR Djibouti OR Ethiopia OR Eritrea OR Kenya OR Réunion OR Rwanda OR Seychelles OR Somalia OR Somaliland OR Tanzania OR Uganda; Western Africa—Benin OR Burkina Faso OR Cape Verde OR Côte D’Ivoire OR Gambia OR Ghana OR Guinea OR Guinea-Bissau OR Liberia OR Mali OR Mauritania OR Niger OR Nigeria OR Senegal OR Sierra Leone OR Togo. See Supplementary Material File S1 for the full literature search terms and combinations that were used on PubMed, Google Scholar, and other databases.

**Figure 1:**
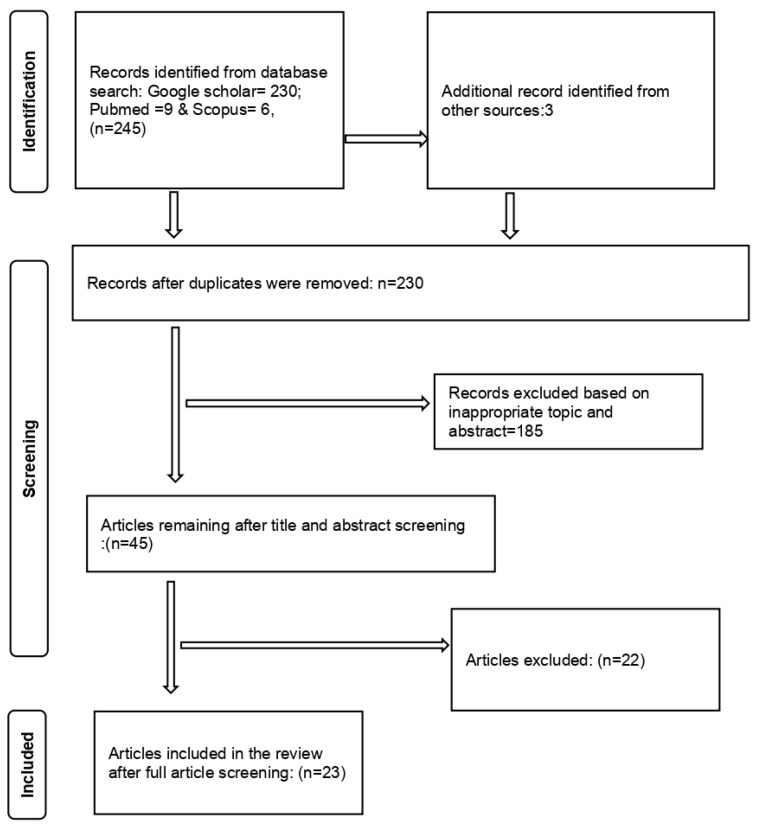
PRISMA diagram of the articles included in the meta-analysis.

The Preferred Reporting Items for Systematic Reviews and Meta-analyses (PRISMA) guidelines were used for the paper selection process (Figure 1). The following were requirements for all articles to be included in this study: (a) studies reporting data from any African country, (b) studies reporting data on fascioliasis infection on intermediate host snails from the Family lymnaeidae (*P. columella*, *R. natalensis,* and *G. truncatula*) to species level, (c) studies reporting distribution of the Family *lymnaeid*ae within Africa, (d) studies that mentioned the diagnostic used in detecting infected snails, (e) only studies written in English, and (f) studies that reported infection in snails that had been sampled from the field and not laboratory-infected snails. Studies without full texts, studies that were review articles, and meta-analyses were excluded. The articles that qualified to be included in the review were retrieved and exported from the different database libraries to the Endnote 20 reference manager.

### 2.2. Data Extraction and Quality Appraisal

The first author’s name, the year of publication, the country of the study, the species of snails used (collection, examination, and infection), the number of snails, as well as the diagnostics used to detect *Fasciola* infection were all included in the data extraction format from the assessed articles. Using the 10 quality-control criteria outlined by the Joanna Briggs Institute Prevalence Critical Appraisal [16], the quality of all included research was evaluated (Supplementary Material File S2).

### 2.3. Data Analysis

The pooled prevalence estimates from the eligible studies were obtained using an inverse variance heterogeneity (IVhet) model in MetaXL version 5.3 (a Microsoft Excel add-in tool for meta-analysis). The IVhet model was chosen above the fixed-effect and random-effect models because, in the absence of heterogeneity, the confidence interval coverage stays relatively close to the nominal level [20,21]. To display the estimated prevalence and its 95% confidence interval, forest plots were created. Cochran’s Q statistic was utilized to gauge the degree of study heterogeneity, and Higgin’s inconsistency statistic (*i*^2^) was used to measure the degree of study variability. When Higgins’s *i*^2^ value is less than 25%, 50%, and 75%, it can be considered that it demonstrates strong homogeneity, medium heterogeneity, and high heterogeneity, respectively [22]. Using the Luis Furuya-Kanamori (LFK) index of the Doi plot, publication bias was evaluated. The size of the LFK index determined the degree of publishing bias. An LFK value within the range of “±1” was considered to be “symmetrical” and classified as the absence of publication bias, while an LFK value outside the range of “±2” was considered to be major asymmetry and high publication bias. Additionally, to explain the observed heterogeneity, subgroup analysis was performed using our data stratified by snail species and the African regions where the studies were conducted [23,24].

## 3. Results

### 3.1. Search Results

The search yielded 245 articles from the different databases, and 15 were identified as duplicates and therefore removed. The 230 remaining articles were screened by title and abstract resulting in the exclusion of 185 articles. Forty-five articles remained after the title and abstract screening. After the full article screening, 22 articles were excluded and 23 articles were considered eligible for inclusion in the systematic review and meta-analysis (Figure 1). The studies included in this review ranged from 1999 to 2022.

The review focused on African countries, but the 23 included articles were from 10 countries (Table 1, Table 2 and Table 3). The method of diagnosis used to detect the *Fasciola* infection was cercarial shedding for 14 articles, 5 articles used both cercarial shedding and crushing, 3 articles used PCR, and 1 article used dissecting.

### 3.2. Infection Rates of Fasciola Intermediate Host Snail Species and Their Distribution in Africa

The highest infection rate was 43.5% (*n* = 10) in Egypt and 17.4% (*n* = 4) in Nigeria. Following was Algeria with 8.7% (*n* = 2), while Zambia, Tanzania, Sudan, Ethiopia, Tunisia, and South Africa all consisted of 4% (*n* = 1). Of the 23 articles, 53.3% (*n* = 14) reported on *R. natalensis* (Table 1); 20% (*n* = 5) reported on *G. truncatula*; and 17% (*n* = 4) of the articles reported on *P. columella.*

**Table 1:** Infection rates of *Radix natalensis* with *Fasciola* spp. in African regions.

African Region	Country	Sample Size	Positive	Infection Rate (%)	Method	Sampling Period	Quality Score	Citation
North Africa	Egypt	1500	1110	74	Cercariae shedding and crushing	April 2016–September 2017	10	[25]
	Egypt	918	197	21.5	Crushing and Cercariae shedding	January 2004–December 2006	10	[26]
	Egypt	917	51	5.5	Cercariae shedding and crushing	Not specified	9	[27]
	Egypt	4939	27	0.6	Crushing technique	March 1997–February 1999	10	[28]
	Sudan	1710	412	23.7	Cercarial shedding	Not specified	9	[29]
	Egypt	2237	91	41	Cercariae shedding	December –August 2005	10	[30]
West Africa	Nigeria	211	18	8.5	Cercarial shedding	January–May 2019	10	[31]
	Nigeria	372	4	1.1	Cercariae shedding	June–December 2014	10	[32]
	Nigeria	2180	12	1.7	Cercariae shedding	August–September 2011	10	[33]
	Nigeria	630	69	10.95	Cercariae shedding	January 2013–December 2013	10	[34]
East Africa	Ethiopia	747	11	1.5	Cercariae shedding	March–May 2016	10	[35]
	Tanzania	832	86	10.3	Cercariae shedding	July 1996–June 1997	10	[36]
	Ethiopia	412	11	2.7	Cercarial shedding	February–May 2016	10	[37]
Southern Africa	Zambia	984	135	13.7	Cercariae shedding	August–October 2003	10	[38]

The information in Table 1 is for *R. natalensis*; it includes the citation of the study the information was extracted from, the country, African region, duration of sampling, sample size, positive snails, infection rate, and the method of diagnosis.

**Figure 2:**
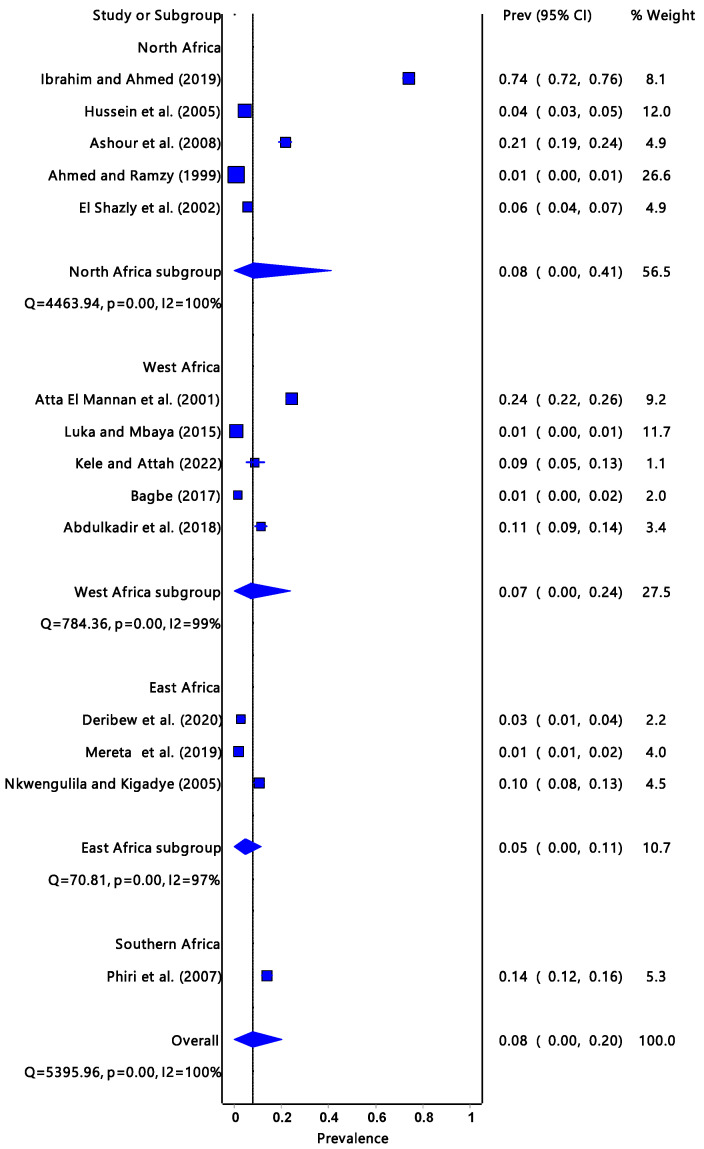
The forest plot represents the prevalence estimate of *Fasciola* intermediate host snail *Radix natalensis* extracted from studies included in the review [25,26,27,28,29,30,31,32,33,34,35,36,37,38].

The pooled prevalence estimate of infectivity for *R. natalensis* (Figure 2) was 8% (95% CI: 0.00–0.20) with a significantly low degree of heterogeneity (I^2^ = 100%, *p* = 0.00). The Southern Africa region had the highest prevalence estimate infectivity at 14% (95% CI: 0.12–0.16), followed by North Africa 8% (95% CI: 0.00–0.41), West Africa 7% (95% CI: 0.00–0.24), and East Africa had the least, which was 5% (95% CI: 0.00–0.11).

#### 3.2.1. Infection Rates of Galba Truncatula in African Regions

A total of 5 articles from the eligible (23) focus on *G. truncatula* sp (Table 2). Interestingly, from the articles obtained, its distribution is restricted to the North African region (Algeria (*n* = 2); Egypt (*n* = 2); and Tunisia (*n*= 1)). The infection rate of the intermediate host ranged between 3.1% and 19.2%; the lowest (3.1%) was recorded in Egypt [27] and the highest (19.2%) was recorded in Tunisia [39].

**Table 2:** Infection rates of *Galba truncatula* with *Fasciola* spp. in African regions.

African Region	Country	Sample Size	Positive Snails	Infection Rate (%)	Method	Sampling Period	Quality Score	Citation
North Africa	Algeria	722	75	10.7	Multiplex PCR	Not specified	9	[40]
	Tunisia	1346	1218	19.2	Cercariae shedding	July 2004–May 2005	10	[39]
	Egypt	731	71	9.7	Crushing technique	March–May 2014	10	[41]
	Egypt	215	7	3.1	Cercariae shedding and crushing	Not specified	9	[27]
	Algeria	1303	888	4.0	Crushing technique	November 2002–May 2003	10	[42]

**Figure 3:**
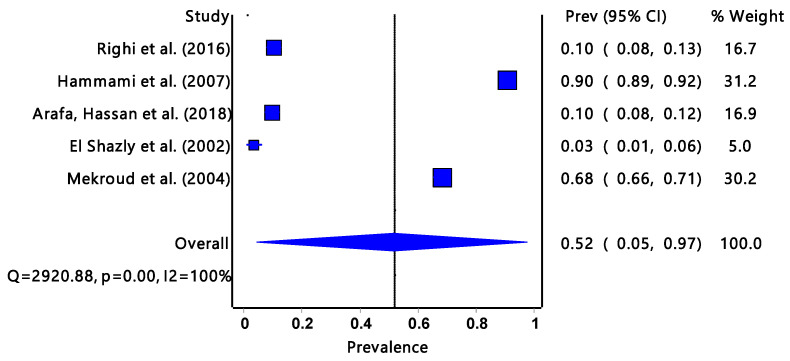
The forest plot represents the pooled prevalence estimate of *Fasciola* intermediate host snail *Galba truncatula* extracted from studies included in the review [27,39,40,41,42].

The pooled prevalence estimated infectivity for *G. truncatula* (Figure 3) was 52% (95% CI: 0.05–0.97) with a significantly low degree of heterogeneity (I^2^ = 100%, *p* = 0.00) and it was only found in the North African region.

#### 3.2.2. Infection Rates of Pseudosuccinea Columella in African Regions

Table 3 shows 4 articles from the 23 eligible. These articles focus on the *P. columella* sp. From the articles obtained, its distribution is limited to the North African region (Egypt (*n* = 3)) and the South African region (South Africa (*n* = 1)). The infection rate of the intermediate host ranged between 0% and 100%; the lowest was recorded in Egypt [27] and the highest (100%) was recorded in South Africa [15].

**Table 3:** Infection rates of *Pseudosuccinea columella* with *Fasciola* spp. in African regions.

African Region	Country	Sample Size	Positive	Infection Rate (%)	Method	Sampling Period	Quality Score	Citation
Southern Africa	South Africa	100	100	100	PCR	November 2017–July 2018	10	[15]
North Africa	Egypt	4087	81	2.0	Crushing technique	March 1997–February 1999	10	[28]
	Egypt	296	38	12.84	PCR	Not specified	9	[43]
	Egypt	45	0	0	Cercariae shedding and crushing	Not specified	9	[27]

**Figure 4:**
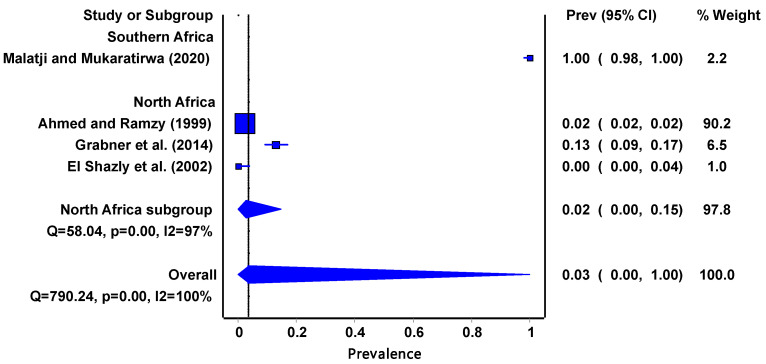
The forest plot represents the pooled prevalence estimate of *Fasciola* intermediate host snail *P. columella* extracted from studies included in the review [15,27,28,43].

The invasive *P. columella* (Figure 4) pooled prevalence estimate of infectivity was 3% (95% CI: 0.00–1.00) and it had a moderate-to-high degree of heterogeneity (I^2^ = 100%, *p* = 0.00). Interestingly the infectivity data were obtained from two regions, Southern Africa at 100% (95%CI: 0.98–1.00) and North Africa at 2% (95% CI: 0.00–0.15).

**Figure 5:**
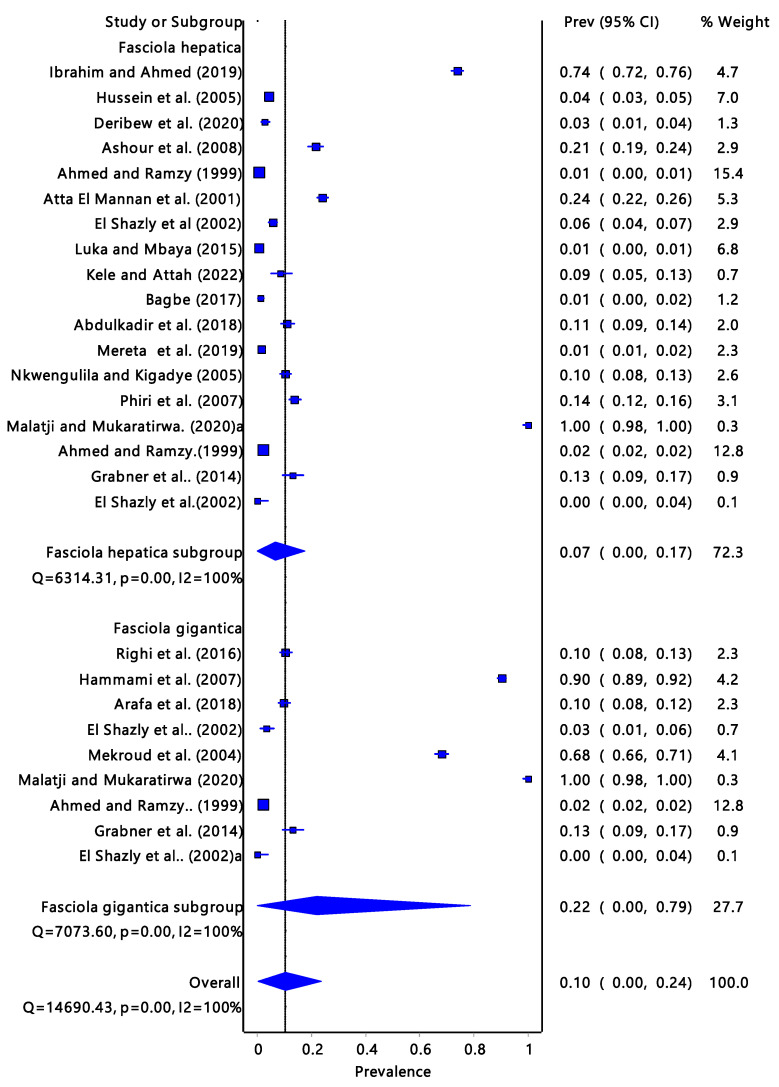
Forest plot represents the pooled prevalence estimate of *Fasciola gigantica* and *Fasciola hepatica* extracted from the studies included in the review [15,25,26,27,28,29,30,31,32,33,34,35,36,37,38,39,40,41,42,43,44].

The *Fasciola* spp. (Figure 5) pooled prevalence estimate of infectivity was 10% (95% CI: 0.00–0.24) and it had a moderate to a high degree of heterogeneity (I^2^= 100%, *p* = 0.00). The infectivity of *F. gigantica* was 7% (95% CI: 0.00–0.17) and *F. hepatica* infectivity was 22% (95% CI: 0.00–0.79).

**Figure 6:**
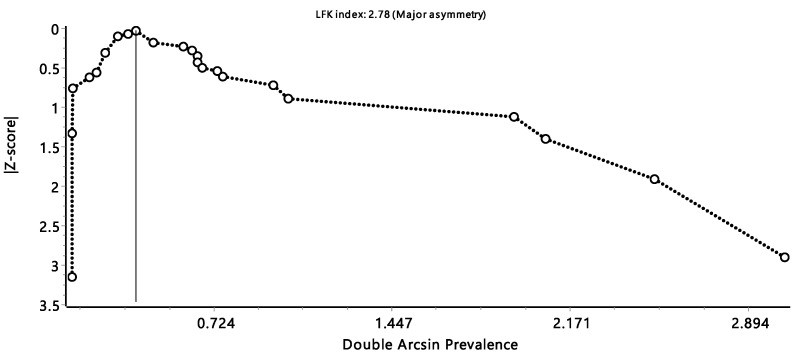
Luis Furuya-Kanamori (LFK) index of the Doi plot presents the publication bias.

The Doi plot showed a significant publication bias, as indicated by the LFK index of 2.78, which indicates a significant asymmetry (Figure 6).

## 4. Discussion

The systematic review and meta-analysis were carried out to determine the infection rate of *Fasciola* intermediate host snails *(P. columella*, *R. natalensis*, and *G. truncatula*) and their distribution in African regions. The distribution of the intermediate host snails and the rate of their infections are indicators of disease hotspots and are hence important to know from a disease control perspective. The findings of this review suggest that naturally infected intermediate host snails (*P. columella*, *R. natalensis*, and *G. truncatula)* are found in various regions of Africa; however, no studies on the infection rates of *Fasciola* intermediate host snails were identified from the Central African region. This may be attributed to conflict in Central African countries which limits research activities; hence, it is not easy to determine infection rates of *Fasciola* intermediate host snails in that region [45].

The native intermediate host snails’ (*R. natalensis* and *G. truncatula*) infectivity prevalence has been studied more than that of invasive *P. columella*. *Radix natalensis* accounts for 60.9% of the studies included in the review which were from various African regions. The Southern Africa region had the highest *Fasciola* infection prevalence but consisted of one study [38], followed by the North Africa region which consisted of studies mainly from Egypt [25,26,27,28,30]. The results of this review showed that Egypt had the highest Fasciola infection prevalence in Africa. These findings are in agreement with Nyindo and Lukambagire [19], who stated that, to date, fascioliasis has been identified in many countries and Egypt has the highest prevalence. *Radix natalensis* had a low overall *Fasciola* infection rate of 8%. There was a wide distribution of *R. natalensis* (North Africa, East Africa, West Africa, and Southern Africa) compared to the other intermediate host snails in this study. Most countries in these regions practice livestock farming and the migration of livestock contributes to the distribution of *Fasciola* spp. The wide distribution of *R. natalensis* was also noted by Malatjli et al. [44], where lymnaeide snail species appeared in 10 of the 12 countries in the East and Southern Africa regions. The results of this review aligned with Moema and Barker [12] who suggested that the primary snail host for *F. hepatica* or *F. gigantica,* common in Africa, is the *R. natalensis* snail. The study by Wamae et al. [46] further stated that the most prevalent liver fluke in sub-Saharan Africa is *F. gigantica*, which thrives in warmer climates probably as a result of the vast distribution of its intermediate host *R. natalensis*.

*Galba truncatula* accounts for 22% (from three countries) of the studies included in the review and it was only found in the North African region with the highest overall infection rate of 52%. Countries in the North African region usually have hot climatic conditions with little rainfall. The study from Tunisia had a significantly high *Fasciola* prevalence rate compared to studies from Algeria and Egypt [11,27,39,40,42]. *Galba truncatula* is an amphibious organism and can survive long dry periods, hence the high percentage of *Fasciola* infection [47]. Temperature is one of the environmental conditions that can affect the host–parasite interaction by compromising immunological processes, thereby lowering host resistance to infections [48].

The results of this review show that there is an overlap of distribution between *R. natalensis* and *G. truncatula* in Africa. According to Malatji et al. [45], overlaps can be local, as in the Nile Delta in Egypt, where the climate is favorable for the coexistence of *Galba* and *Radix* species throughout the year, or zonal, as in highland areas where the cold-mild weather favors *F. hepatica* and *G. truncatula* and the lowlands provide the warm-hot climate for *Radix* spp. and the *F. gigantica* system. This latter type of overlap has been described [47].

The invasive *P. columella* accounts for 17% of the studies included in the review which yielded an overall infection rate of 3%. The prevalence of *Fasciola* infectivity in *P. columella* may be affected by biological factors such as predators and competitors because it is still adapting in the African regions. Predators prey on freshwater snails and consume trematode larvae, miracidia. Consequently, the prevalence of freshwater snail infection with trematodes may be reduced. The distribution and infectivity of these *Fasciola* intermediate host snails may be further complicated by climate change. Hence, there is need for more studies on the impact of climate change on their presence, abundance, co-habitation, and infectivity.

In Africa, the infection rate of *Fasciola* spp. in the definitive hosts is significantly much more studied than in the intermediate host snails. It is important to note that the infection rate of ruminants can be significantly higher than that of intermediate hosts. One of the reasons is that it is easier to deal with livestock than to deal with snails whose abundance and distribution are not easy to estimate. Livestock are of more economic importance than intermediate hosts. There were reports about fascioliasis in ruminants from Chad, Egypt, Ethiopia, Kenya, Nigeria, Sudan, Tanzania, Tunisia, Zambia, and Zimbabwe. The highest number of reports were recorded in Kenya and Ethiopia. According to Jones et al. [49], the majority of infections are caused by bovine fascioliasis, which is widely distributed throughout the world and accounts for 29% of zoonoses. The infection rate of *Fasciola* spp. in the intermediate host snails may give an indication of the presence and burden of the disease in the community, i.e., livestock and humans. El Shazly et al. [27] examined all three intermediate host snails which were co-existing in Egypt. *Radix natalensis, G. truncatula,* and *P. columella* had infection rates of 5.5%, 3.1%, and 0%, respectively. The results from this review (Figure 5) suggested that *F. hepatica* has the highest infection prevalence at 22% compared to *F. gigantica* at 7%; however, the overall pooled prevalence was low. These findings were in line with the results (Figure 2, Figure 3 and Figure 4) obtained from the *Fasciola* infection prevalence of intermediate host snails of these *Fasciola* spp. In this study. Generally, the prevalence was fairly low. According to Bowden [50], a hospital in Egypt received patients with fever of unknown origin, and 4% of them tested positive for *F. hepatica*. Previously endemic in isolated foci along the Nile River in Egypt, the *F. hepatica*-caused disease is now widespread throughout the Nile valley. According to an earlier study by Esteban et al. [51] that looked at parasite causes of pediatric hepatomegaly, about 8.7% of the patients under investigation had fascioliasis in the villages in the Nile Delta of Egypt. This is an indication of the consequences of the transmission of *Fasciola*.

The main limitation of this review is that the cercarial shedding method was used in 70% (16/23) of the studies included in the review to determine *Fasciola* infections. Due to its accessibility and affordability, this is a frequent diagnostic technique for identifying infections, and the cercarial shedding technique is time- and labor-intensive; it is known to significantly underestimate the true prevalence of infection in the intermediate host snail. The low pooled prevalence in this meta-analysis may therefore be partially attributable to the infection detection method [52]. A significant number of reviews in Africa focus on the infection rate of *Fasciola* in definitive hosts [53,54,55,56], and according to our knowledge, there has not been a review focusing on the infection rate of intermediate host snails in all the regions of Africa; hence, the need of this review is significant.

## 5. Conclusions

In conclusion, this review has revealed that naturally infected Lymnaeide intermediate host snails are distributed in the various African regions and the *Fasciola* infection prevalence varies in the different regions depending on the IH and *Fasciola* spp. Overall, the low number of studies retrieved shows that the role of intermediate host snails in the transmission of *Fasciola* spp. is still neglected in Africa. This calls for more studies on the comparative analysis of the environmental and climatic factors related to the presence, abundance, co-habitation, and infection rates of these *Fasciola* intermediate host snails and to elucidate the potential impacts of climate change on the transmission dynamics of this disease in Africa. Our findings also emphasize the need for more malacology surveys using enhanced infectivity diagnostic methods throughout the various African regions to improve the diagnosis of infection and integrated snail control strategies to prevent the spread of fascioliasis.

## Data Availability

Not applicable.

## Supplementary Materials

The following supporting information can be downloaded at: https://www.mdpi.com/article/10.3390/tropicalmed8100467/s1, File S1: Search strategy; File S2: Quality assessment.

## References

[1] Beesley N., Caminade C., Charlier J., Flynn R., Hodgkinson J., Martinez-Moreno A., Martinez-Valladares M., Perez J., Rinaldi L., Williams D. (2018). Fasciola and fasciolosis in ruminants in Europe: Identifying research needs. Transbound. Emerg. Dis..

[2] Haydock L., Pomroy W., Stevenson M., Lawrence K. (2016). A growing degree-day model for determination of *Fasciola hepatica* infection risk in New Zealand with future predictions using climate change models. Vet. Parasitol..

[3] Dida G.O., Gelder F.B., Anyona D.N., Matano A.-S., Abuom P.O., Adoka S.O., Ouma C., Kanangire C.K., Owuor P.O., Ofulla A.V. (2014). Distribution and abundance of schistosomiasis and fascioliasis host snails along the Mara River in Kenya and Tanzania. Infect. Ecol. Epidemiol..

[4] Pal M., Abdurahman M., Zewdu M. (2014). Growing significance of fascioliasis as an emerging zoonosis. Ethiop. Int. J. Multidiscip. Res..

[5] Black C.L., Mwinzi P.N., Muok E.M., Abudho B., Fitzsimmons C.M., Dunne D.W., Karanja D.M., Secor W.E., Colley D.G. (2010). Influence of exposure history on the immunology and development of resistance to human Schistosomiasis mansoni. PLoS Negl. Trop. Dis..

[6] Sabourin E., Alda P., Vázquez A., Hurtrez-Boussès S., Vittecoq M. (2018). Impact of human activities on fasciolosis transmission. Trends Parasitol..

[7] Charlier J., Ghebretinsae A.H., Levecke B., Ducheyne E., Claerebout E., Vercruysse J. (2016). Climate-driven longitudinal trends in pasture-borne helminth infections of dairy cattle. Int. J. Parasitol..

[8] Pozio E. (2020). How globalization and climate change could affect foodborne parasites. Exp. Parasitol..

[9] Mas-Coma S., Valero M.A., Bargues M.D. (2014). Fascioliasis. Digenetic Trematodes.

[10] Sripa J., Kiatsopit N., Piratae S. (2016). Prevalence of trematode larvae in intermediate hosts: Snails and fish in Ko Ae sub-district of Khueang Nai, Ubon Ratchathani province, Thailand. Southeast Asian J. Trop. Med. Public Health.

[11] Arafa W.M. (2015). Detection of *Fasciola hepatica* infection in cattle and *Lymnaea truncatula* snails in Dakhla Oasis, Egypt. Egypt. Vet. Med. Soc. Parasitol. J..

[12] Moema E., King P., Baker C. (2008). Cercariae developing in Lymnaea natalensis Krauss, 1848 collected in the vicinity of Pretoria, Gauteng Province, South Africa. Onderstepoort J. Vet. Res..

[13] De Kock K., Wolmarans C., Bornman M. (2003). Distribution and habitats of the snail Lymnaea truncatula, intermediate host of the liver fluke *Fasciola hepatica*, in South Africa. J. S. Afr. Vet. Assoc..

[14] Dillon R.T. (2000). The Ecology of Freshwater Molluscs.

[15] Malatji M., Mukaratirwa S. (2020). Molecular detection of natural infection of *Lymnaea* (*Pseudosuccinea*) *columella* (Gastropoda: Lymnaeidae) with *Fasciola gigantica* (Digenea: Fasciolidae) from two provinces of South Africa. J. Helminthol..

[16] Mage C., Bourgne H., Toullieu J.-M., Rondelaud D., Dreyfuss G. (2002). Fasciola hepatica and Paramphistomum daubneyi: Changes in prevalences of natural infections in cattle and in *Lymnaea truncatula* from central France over the past 12 years. Vet. Res..

[17] Cucher M., Carnevale S., Prepelitchi L., Labbé J., Wisnivesky-Colli C. (2006). PCR diagnosis of Fasciola hepatica in field-collected *Lymnaea columella* and *Lymnaea viatrix* snails. Vet. Parasitol..

[18] Malatji M., Lamb J., Mukaratirwa S. (2019). Molecular characterization of liver fluke intermediate host lymnaeids (Gastropoda: Pulmonata) snails from selected regions of Okavango Delta of Botswana, KwaZulu-Natal and Mpumalanga provinces of South Africa. Vet. Parasitol. Reg. Stud. Rep..

[19] Nyindo M., Lukambagire A.-H. (2015). Fascioliasis: An ongoing zoonotic trematode infection. BioMed Res. Int..

[20] Doi S.A., Barendregt J.J., Khan S., Thalib L., Williams G.M. (2015). Advances in the meta-analysis of heterogeneous clinical trials I: The inverse variance heterogeneity model. Contemp. Clin. Trials.

[21] Doi S.A., Furuya-Kanamori L. (2020). Selecting the best meta-analytic estimator for evidence-based practice: A simulation study. JBI Evid. Implement..

[22] Ahn E., Kang H. (2018). Introduction to systematic review and meta-analysis. Korean J. Anesthesiol..

[23] Ioannidis J.P. (2008). Interpretation of tests of heterogeneity and bias in meta-analysis. J. Eval. Clin. Pract..

[24] Higgins J.P., Thompson S.G., Deeks J.J., Altman D.G. (2003). Measuring inconsistency in meta-analyses. BMJ.

[25] Ibrahim A.M., Ahmed A.K. (2019). Trematode cercarial fauna obtained from the field-collected freshwater snails *Lymnaea natalensis* in Egypt. Bull. Natl. Res. Cent..

[26] Ashour A., Mostafa B., Taha H., Azzam A. (2008). Distribution of Natural Populations of *Lymnaea* Snails in Some Egyptian Governorates and Their Natural Infection with *Fasciola*. J. Environ. Sci..

[27] El Shazly A.M., Helmy M.M., Haridy F.M., El Sharkawy E.M., Morsy T.A. (2002). Fasciola immature stages sought in Lymnaea species and Biomphalaria species in the water bodies of Dakahlia Governorate. J.-Egypt. Soc. Parasitol..

[28] Ahmed A.H., Ramzy R.M. (1999). Infection of two lymnaeid snails with Fasciola gigantica in Giza, a field study. J.-Egypt. Soc. Parasitol..

[29] Atta El Mannan A., Bushara H., Majid A. (2001). Some aspects of the epidemiology of bovine fasciolosis in northern Gazira and Khartoum State. Sudan J. Vet. Res..

[30] Hussein A., Califa R., Mas-Coma S. (2005). Trematode larval stages infecting Radix natalensis (Gastropoda: Lymnaeidae) in Qena Governorate, Egypt, with special reference to fasciolid cercariae. Res. Rev. Parasitol..

[31] Kele M., Attah D.D. (2022). Cercarial shedding and trematodes infestation in freshwater snail species from selected freshwater bodies in Zuru Emirate, Kebbi State, Nigeria. Int. J. Biomed. Health Sci..

[32] Bagbe A.S. (2017). Malacological study of snail intermediate hosts of trematode parasites in Okitipupa Local Government Area, Ondo State, Nigeria. Acad. J..

[33] Luka J., Mbaya A.W. (2015). Cercarial shedding of trematodes and their associated snail intermediate hosts in Borno State, Nigeria. Asian Pac. J. Trop. Dis..

[34] Abdulkadir F.M., Maikaje D., Umar Y. (2018). Cercarial Diversity in Freshwater Snails from Selected Freshwater Bodies and Its Implication for Veterinary and Public Health in Kaduna State, Nigeria. Int. J. Anim. Vet. Sci..

[35] Mereta S.T., Bedewi J., Yewhalaw D., Mandefro B., Abdie Y., Tegegne D., Birke W., Mulat W.L., Kloos H. (2019). Environmental determinants of distribution of freshwater snails and trematode infection in the Omo Gibe River Basin, southwest Ethiopia. Infect. Dis. Poverty.

[36] Nkwengulila G., Kigadye E. (2005). Occurrence of digenean larvae in freshwater snails in the Ruvu Basin, Tanzania. Tanzan. J. Sci..

[37] Deribew K., Jaleta E., Mandefro B., Mekonnen Z., Yewhalaw D., Abdie Y., Mereta S.T. (2020). Effects of land use on intermediate snail host fauna, abundance, distribution and cercariae infection rate in Omo-Gibe river basin, Ethiopia. Res. Sq..

[38] Phiri A., Phiri I., Chota A., Monrad J. (2007). Trematode infections in freshwater snails and cattle from the Kafue wetlands of Zambia during a period of highest cattle–water contact. J. Helminthol..

[39] Hammami H., Hamed N., Ayadi A. (2007). Epidemiological studies on Fasciola hepatica in Gafsa Oases (south west of Tunisia). Parasite.

[40] Righi S., Benakhla A., Mekroud A., Ouchene N., Sedraoui S. (2016). Prevalence of *Fasciola hepatica* in *Galba truncatula* detected by multiplex PCR in the province of El Tarf (Algeria). Trop. Biomed..

[41] Arafa W., Hassan A., Snousi S., El-Dakhly K.M., Holman P., Craig T., Aboelhadid S. (2018). *Fasciola hepatica* infections in cattle and the freshwater snail *Galba truncatula* from Dakhla Oasis, Egypt. J. Helminthol..

[42] Mekroud A., Benakhla A., Vignoles P., Rondelaud D., Dreyfuss G. (2004). Preliminary studies on the prevalences of natural fasciolosis in cattle, sheep, and the host snail (*Galba truncatula*) in north-eastern Algeria. Parasitol. Res..

[43] Grabner D., Mohamed F., Nachev M., Méabed E., Sabry A. (2014). Invasion Biology Meets Parasitology: A Case Study of Parasite Spill-Back. PloS ONE.

[44] Malatji M., Pfukenyi D., Mukaratirwa S. (2020). *Fasciola* species and their vertebrate and snail intermediate hosts in East and Southern Africa: A review. J. Helminthol..

[45] Nukeri S., Malatji M.P., Sengupta M.E., Vennervald B.J., Stensgaard A.-S., Chaisi M., Mukaratirwa S. (2022). Potential Hybridization of *Fasciola hepatica* and *F. gigantica* in Africa—A Scoping Review. Pathogens.

[46] Wamae L., Hammond J., Harrison L., Onyango-Abuje J. (1998). Comparison of production losses caused by chronic Fasciola gigantica infection in yearling Friesian and Boran cattle. Trop. Anim. Health Prod..

[47] Mas-Coma S., Valero M.A., Bargues M.D. (2009). Climate change effects on trematodiases, with emphasis on zoonotic fascioliasis and schistosomiasis. Vet. Parasitol..

[48] Martinez J., Merino S. (2011). Host-parasite interactions under extreme climatic conditions. Current Zoology.

[49] Jones K.E., Patel N.G., Levy M.A., Storeygard A., Balk D., Gittleman J.L., Daszak P. (2008). Global trends in emerging infectious diseases. Nature.

[50] Bowden L. (2008). Fascioliasis and fasciolopsiasis: Similar names, similar diseases. J. Spec. Oper. Med..

[51] Esteban J.-G., Gonzalez C., Curtale F., Muñoz-Antoli C., Valero M.A., Bargues M.D., El Sayed M., El Wakeel A.A., Abdel-Wahab Y., Montresor A. (2003). Hyperendemic fascioliasis associated with schistosomiasis in villages in the Nile Delta of Egypt. Am. J. Trop. Med. Hyg..

[52] Caron Y., Rondelaud D., Losson B. (2008). The detection and quantification of a digenean infection in the snail host with special emphasis on *Fasciola* sp.. Parasitol. Res..

[53] Elelu N., Eisler M. (2018). A review of bovine fasciolosis and other trematode infections in Nigeria. J. Helminthol..

[54] Mehmood K., Zhang H., Sabir A.J., Abbas R.Z., Ijaz M., Durrani A.Z., Saleem M.H., Rehman M.U., Iqbal M.K., Wang Y. (2017). A review on epidemiology, global prevalence and economical losses of fasciolosis in ruminants. Microb. Pathog..

[55] Alemu B. (2019). Bovine Fasciolosis in Ethiopia—A review. J. Vet. Anim. Res..

[56] Tsega M. (2015). A review on ruminant fasciolosis. Open Access Libr. J..

