# Supplementary material for: Infection Rates of Fasciola Intermediate Host Snail Species and Their Distribution in Africa: A Systematic Review and Meta-Analysis
Source: Trop Med Infect Dis. 2023 Oct 6;8(10):467. doi: 10.3390/tropicalmed8100467 (PMC10610779; doi:10.3390/tropicalmed8100467)
Supplement: Supplementary file 1 [file tropicalmed-08-00467-s001.zip › File S1 Search strategy.pdf]

Search Details

("fascioliasis"[MeSH Terms] OR "fascioliasis"[All Fields] OR ("fasciola hepatica"[MeSH Terms] OR ("fasciola"[All Fields] AND "hepatica"[All Fields]) OR "fasciola hepatica"[All Fields] OR ("liver"[All Fields] AND "fluke"[All Fields]) OR "liver fluke"[All Fields]) OR (("fasciola"[MeSH Terms] OR "fasciola"[All Fields]) AND "gigantica"[All Fields]) OR ("fasciola hepatica"[MeSH Terms] OR ("fasciola"[All Fields] AND "hepatica"[All Fields]) OR "fasciola hepatica"[All Fields]) OR ("fasciola"[MeSH Terms] OR "fasciola"[All Fields] OR ("fasciola"[All Fields] AND "sp"[All Fields]) OR "fasciola sp"[All Fields])) AND ("infect"[All Fields] OR "infectability"[All Fields] OR "infectable"[All Fields] OR "infectant"[All Fields] OR "infectants"[All Fields] OR "infected"[All Fields] OR "infecteds"[All Fields] OR "infectibility"[All Fields] OR "infectible"[All Fields] OR "infecting"[All Fields] OR "infection s"[All Fields] OR "infections"[MeSH Terms] OR "infections"[All Fields] OR "infection"[All Fields] OR "infective"[All Fields] OR "infectiveness"[All Fields] OR "infectives"[All Fields] OR "infectivities"[All Fields] OR "infects"[All Fields] OR "pathogenicity"[MeSH Subheading] OR "pathogenicity"[All Fields] OR "infectivity"[All Fields] OR (("infect"[All Fields] OR "infectability"[All Fields] OR "infectable"[All Fields] OR "infectant"[All Fields] OR "infectants"[All Fields] OR "infected"[All Fields] OR "infecteds"[All Fields] OR "infectibility"[All Fields] OR "infectible"[All Fields] OR "infecting"[All Fields] OR "infection s"[All Fields] OR "infections"[MeSH Terms] OR "infections"[All Fields] OR "infection"[All Fields] OR "infective"[All Fields] OR "infectiveness"[All Fields] OR "infectives"[All Fields] OR "infectivities"[All Fields] OR "infects"[All Fields] OR "pathogenicity"[MeSH Subheading] OR "pathogenicity"[All Fields] OR "infectivity"[All Fields]) AND ("j rehabil assist technol eng"[Journal] OR "rate"[All Fields])) OR ("intense"[All Fields] OR "intensely"[All Fields] OR "intensities"[All Fields] OR "intensity"[All Fields] OR "intensively"[All Fields]) OR ("epidemiology"[MeSH Subheading] OR "epidemiology"[All Fields] OR "prevalence"[All Fields] OR "prevalence"[MeSH Terms] OR "prevalance"[All Fields] OR "prevalences"[All Fields] OR "prevalence s"[All Fields] OR "prevalent"[All Fields] OR "prevalently"[All Fields] OR "prevalents"[All Fields]) OR ("epidemiology"[MeSH Subheading] OR "epidemiology"[All Fields] OR "incidence"[All Fields] OR "incidence"[MeSH Terms] OR "incidences"[All Fields] OR "incident"[All Fields] OR "incidents"[All Fields])) AND ((("lymnaea"[MeSH Terms] OR "lymnaea"[All Fields] OR "lymnaeas"[All Fields]) AND ("columella"[All Fields] OR "columellae"[All Fields] OR "columellas"[All Fields])) OR (("lymnaea"[MeSH Terms] OR "lymnaea"[All Fields] OR "lymnaeas"[All Fields]) AND "natalensis"[All Fields]) OR (("lymnaea"[MeSH Terms] OR "lymnaea"[All Fields] OR "lymnaeas"[All Fields]) AND "truncatula"[All Fields]) OR ("Pseudosuccinea"[All Fields] AND ("columella"[All Fields] OR "columellae"[All Fields] OR "columellas"[All Fields])) OR (("plant roots"[MeSH Terms] OR ("plant"[All Fields] AND "roots"[All Fields]) OR "plant roots"[All Fields] OR "radix"[All Fields] OR "radixes"[All Fields]) AND "natalensis"[All Fields]) OR ("Galba"[All Fields] AND "truncatula"[All Fields])) AND ((("fasciola"[MeSH Terms] OR "fasciola"[All Fields]) AND ("intermediate"[All Fields] OR "intermediated"[All Fields] OR "intermediately"[All Fields] OR "intermediates"[All Fields]) AND "host"[All Fields]) OR (("intermediate"[All Fields] OR "intermediated"[All Fields] OR "intermediately"[All Fields] OR "intermediates"[All Fields]) AND "host"[All Fields] AND ("snail s"[All Fields] OR "snails"[MeSH Terms] OR "snails"[All Fields] OR "snail"[All Fields])) OR (("fresh water"[MeSH Terms] OR ("fresh"[All Fields] AND "water"[All Fields]) OR "fresh water"[All Fields] OR "freshwater"[All Fields] OR "freshwaters"[All Fields]) AND ("snail s"[All Fields] OR "snails"[MeSH Terms] OR "snails"[All Fields] OR "snail"[All Fields])) OR (("snail s"[All Fields] OR "snails"[MeSH Terms] OR "snails"[All Fields] OR "snail"[All Fields]) AND ("genetic vectors"[MeSH Terms] OR ("genetic"[All Fields] AND "vectors"[All Fields]) OR "genetic vectors"[All Fields] OR "vector"[All Fields] OR "vectors"[All Fields] OR "vector s"[All Fields] OR "vectored"[All Fields] OR "vectoring"[All Fields] OR "vectorization"[All Fields] OR "vectorize"[All Fields] OR "vectorized"[All Fields] OR "vectorizing"[All

Fields])) OR ("Malacology"[All Fields] AND ("survey s"[All Fields] OR "surveyed"[All Fields] OR "surveying"[All Fields] OR "surveys and questionnaires"[MeSH Terms] OR ("surveys"[All Fields] AND "questionnaires"[All Fields]) OR "surveys and questionnaires"[All Fields] OR "survey"[All Fields] OR "surveys"[All Fields]))) AND ((("algeria"[MeSH Terms] OR "algeria"[All Fields] OR ("egypt"[MeSH Terms] OR "egypt"[All Fields] OR "egypt s"[All Fields]) OR ("libya"[MeSH Terms] OR "libya"[All Fields]) OR "Morroco"[All Fields] OR ("sudan"[MeSH Terms] OR "sudan"[All Fields] OR "sudans"[All Fields] OR "sudan s"[All Fields]) OR ("tunisia"[MeSH Terms] OR "tunisia"[All Fields]) OR ("angola"[MeSH Terms] OR "angola"[All Fields] OR "angola s"[All Fields]) OR ("cameroon"[MeSH Terms] OR "cameroon"[All Fields] OR "cameroons"[All Fields] OR "cameroon s"[All Fields]) OR ("central african republic"[MeSH Terms] OR ("central"[All Fields] AND "african"[All Fields] AND "republic"[All Fields]) OR "central african republic"[All Fields]) OR ("chad"[MeSH Terms] OR "chad"[All Fields]) OR (("congo"[MeSH Terms] OR "congo"[All Fields]) AND ("republic"[All Fields] OR "republic s"[All Fields] OR "republics"[All Fields])) OR "Brazzaville"[All Fields] OR (("democrat"[All Fields] OR "democratic"[All Fields] OR "democratically"[All Fields] OR "democratization"[All Fields] OR "democratize"[All Fields] OR "democratized"[All Fields] OR "democratizing"[All Fields] OR "democrats"[All Fields]) AND ("republic"[All Fields] OR "republic s"[All Fields] OR "republics"[All Fields]) AND ("congo"[MeSH Terms] OR "congo"[All Fields])) OR ("equatorial guinea"[MeSH Terms] OR ("equatorial"[All Fields] AND "guinea"[All Fields]) OR "equatorial guinea"[All Fields]) OR ("gabon"[MeSH Terms] OR "gabon"[All Fields]) OR ("Sao"[All Fields] AND "Tome"[All Fields]) OR ("Sao"[All Fields] AND "Tome"[All Fields] AND ("principe"[All Fields] OR "principes"[All Fields])) OR ("botswana"[MeSH Terms] OR "botswana"[All Fields] OR "botswana s"[All Fields]) OR ("lesotho"[MeSH Terms] OR "lesotho"[All Fields]) OR ("namibia"[MeSH Terms] OR "namibia"[All Fields] OR "namibia s"[All Fields]) OR ("south africa"[MeSH Terms] OR ("south"[All Fields] AND "africa"[All Fields]) OR "south africa"[All Fields]) OR ("zimbabwe"[MeSH Terms] OR "zimbabwe"[All Fields] OR "zimbabwe s"[All Fields]) OR ("zambia"[MeSH Terms] OR "zambia"[All Fields] OR "zambia s"[All Fields]) OR ("mozambique"[MeSH Terms] OR "mozambique"[All Fields] OR "mozambique s"[All Fields]) OR ("mauritius"[MeSH Terms] OR "mauritius"[All Fields]) OR ("eswatini"[MeSH Terms] OR "eswatini"[All Fields] OR "swaziland"[All Fields]) OR ("madagascar"[MeSH Terms] OR "madagascar"[All Fields] OR "madagascar s"[All Fields]) OR ("mauritius"[MeSH Terms] OR "mauritius"[All Fields]) OR ("burundi"[MeSH Terms] OR "burundi"[All Fields]) OR ("comoros"[MeSH Terms] OR "comoros"[All Fields] OR "comoro"[All Fields]) OR ("djibouti"[MeSH Terms] OR "djibouti"[All Fields]) OR ("ethiopia"[MeSH Terms] OR "ethiopia"[All Fields] OR "ethiopia s"[All Fields]) OR ("eritrea"[MeSH Terms] OR "eritrea"[All Fields]) OR ("kenya"[MeSH Terms] OR "kenya"[All Fields] OR "kenya s"[All Fields]) OR ("rwanda"[MeSH Terms] OR "rwanda"[All Fields] OR "rwanda s"[All Fields]) OR ("seychelles"[MeSH Terms] OR "seychelles"[All Fields]) OR ("somalia"[MeSH Terms] OR "somalia"[All Fields] OR "somalia s"[All Fields]) OR ("tanzania"[MeSH Terms] OR "tanzania"[All Fields] OR "tanzania s"[All Fields]) OR ("uganda"[MeSH Terms] OR "uganda"[All Fields] OR "uganda s"[All Fields]) OR ("benin"[MeSH Terms] OR "benin"[All Fields] OR "benin s"[All Fields])) AND ("burkina faso"[MeSH Terms] OR ("burkina"[All Fields] AND "faso"[All Fields]) OR "burkina faso"[All Fields])) OR ("cabo verde"[MeSH Terms] OR ("cabo"[All Fields] AND "verde"[All Fields]) OR "cabo verde"[All Fields] OR ("cape"[All Fields] AND "verde"[All Fields]) OR "cape verde"[All Fields]) OR ("cote d ivoire"[MeSH Terms] OR ("cote"[All Fields] AND "d ivoire"[All Fields]) OR "cote d ivoire"[All Fields]) OR ("gambia"[MeSH Terms] OR "gambia"[All Fields] OR "gambia s"[All Fields]) OR ("ghana"[MeSH Terms] OR "ghana"[All Fields] OR "ghana s"[All Fields]) OR ("guinea"[MeSH Terms] OR "guinea"[All Fields] OR "guinea s"[All Fields] OR "guineas"[All Fields]) OR ("guinea bissau"[MeSH Terms] OR "guinea bissau"[All Fields] OR ("guinea"[All Fields] AND "bissau"[All Fields]) OR "guinea bissau"[All Fields]) OR ("liberia"[MeSH Terms] OR "liberia"[All Fields] OR "liberia s"[All Fields]) OR ("mali"[MeSH Terms] OR "mali"[All Fields]) OR ("mauritania"[MeSH Terms] OR "mauritania"[All Fields]) OR ("niger"[MeSH Terms] OR "niger"[All Fields]) OR

("nigeria"[MeSH Terms] OR "nigeria"[All Fields] OR "nigeria s"[All Fields]) OR (("sierra"[All Fields] OR "sierras"[All Fields]) AND "Eone"[All Fields]) OR ("sierra leone"[MeSH Terms] OR ("sierra"[All Fields] AND "leone"[All Fields]) OR "sierra leone"[All Fields]) OR ("togo"[MeSH Terms] OR "togo"[All Fields]))

("fascioliasis"[MeSH Terms] OR "fascioliasis"[All Fields] OR ("fasciola hepatica"[MeSH Terms] OR ("fasciola"[All Fields] AND "hepatica"[All Fields]) OR "fasciola hepatica"[All Fields] OR ("liver"[All Fields] AND "fluke"[All Fields]) OR "liver fluke"[All Fields]) OR (("fasciola"[MeSH Terms] OR "fasciola"[All Fields]) AND "gigantica"[All Fields]) OR ("fasciola hepatica"[MeSH Terms] OR ("fasciola"[All Fields] AND "hepatica"[All Fields]) OR "fasciola hepatica"[All Fields]) OR ("fasciola"[MeSH Terms] OR "fasciola"[All Fields] OR ("fasciola"[All Fields] AND "sp"[All Fields]) OR "fasciola sp"[All Fields])) AND ("infect"[All Fields] OR "infectability"[All Fields] OR "infectable"[All Fields] OR "infectant"[All Fields] OR "infectants"[All Fields] OR "infected"[All Fields] OR "infecteds"[All Fields] OR "infectibility"[All Fields] OR "infectible"[All Fields] OR "infecting"[All Fields] OR "infection s"[All Fields] OR "infections"[MeSH Terms] OR "infections"[All Fields] OR "infection"[All Fields] OR "infective"[All Fields] OR "infectiveness"[All Fields] OR "infectives"[All Fields] OR "infectivities"[All Fields] OR "infects"[All Fields] OR "pathogenicity"[MeSH Subheading] OR "pathogenicity"[All Fields] OR "infectivity"[All Fields] OR (("infect"[All Fields] OR "infectability"[All Fields] OR "infectable"[All Fields] OR "infectant"[All Fields] OR "infectants"[All Fields] OR "infected"[All Fields] OR "infecteds"[All Fields] OR "infectibility"[All Fields] OR "infectible"[All Fields] OR "infecting"[All Fields] OR "infection s"[All Fields] OR "infections"[MeSH Terms] OR "infections"[All Fields] OR "infection"[All Fields] OR "infective"[All Fields] OR "infectiveness"[All Fields] OR "infectives"[All Fields] OR "infectivities"[All Fields] OR "infects"[All Fields] OR "pathogenicity"[MeSH Subheading] OR "pathogenicity"[All Fields] OR "infectivity"[All Fields]) AND ("j rehabil assist technol eng"[Journal] OR "rate"[All Fields])) OR ("intense"[All Fields] OR "intensely"[All Fields] OR "intensities"[All Fields] OR "intensity"[All Fields] OR "intensively"[All Fields]) OR ("epidemiology"[MeSH Subheading] OR "epidemiology"[All Fields] OR "prevalence"[All Fields] OR "prevalence"[MeSH Terms] OR "prevalance"[All Fields] OR "prevalences"[All Fields] OR "prevalence s"[All Fields] OR "prevalent"[All Fields] OR "prevalently"[All Fields] OR "prevalents"[All Fields]) OR ("epidemiology"[MeSH Subheading] OR "epidemiology"[All Fields] OR "incidence"[All Fields] OR "incidence"[MeSH Terms] OR "incidences"[All Fields] OR "incident"[All Fields] OR "incidents"[All Fields])) AND ((("lymnaea"[MeSH Terms] OR "lymnaea"[All Fields] OR "lymnaeas"[All Fields]) AND ("columella"[All Fields] OR "columellae"[All Fields] OR "columellas"[All Fields])) OR (("lymnaea"[MeSH Terms] OR "lymnaea"[All Fields] OR "lymnaeas"[All Fields]) AND "natalensis"[All Fields]) OR (("lymnaea"[MeSH Terms] OR "lymnaea"[All Fields] OR "lymnaeas"[All Fields]) AND "truncatula"[All Fields]) OR ("Pseudosuccinea"[All Fields] AND ("columella"[All Fields] OR "columellae"[All Fields] OR "columellas"[All Fields])) OR (("plant roots"[MeSH Terms] OR ("plant"[All Fields] AND "roots"[All Fields]) OR "plant roots"[All Fields] OR "radix"[All Fields] OR "radixes"[All Fields]) AND "natalensis"[All Fields]) OR ("Galba"[All Fields] AND "truncatula"[All Fields])) AND ((("fasciola"[MeSH Terms] OR "fasciola"[All Fields]) AND ("intermediate"[All Fields] OR "intermediated"[All Fields] OR "intermediately"[All Fields] OR "intermediates"[All Fields]) AND "host"[All Fields]) OR (("intermediate"[All Fields] OR "intermediated"[All Fields] OR "intermediately"[All Fields] OR "intermediates"[All Fields]) AND "host"[All Fields] AND ("snail s"[All Fields] OR "snails"[MeSH Terms] OR "snails"[All Fields] OR "snail"[All Fields])) OR (("fresh water"[MeSH Terms] OR ("fresh"[All Fields] AND "water"[All Fields]) OR "fresh water"[All Fields] OR "freshwater"[All Fields] OR "freshwaters"[All Fields]) AND ("snail s"[All Fields] OR "snails"[MeSH Terms] OR "snails"[All Fields] OR "snail"[All Fields])) OR (("snail s"[All Fields] OR "snails"[MeSH Terms] OR "snails"[All Fields] OR "snail"[All Fields]) AND ("genetic vectors"[MeSH Terms] OR ("genetic"[All Fields] AND "vectors"[All Fields]) OR "genetic vectors"[All Fields] OR "vector"[All Fields] OR

"vectors"[All Fields] OR "vector s"[All Fields] OR "vectored"[All Fields] OR "vectoring"[All Fields] OR "vectorization"[All Fields] OR "vectorize"[All Fields] OR "vectorized"[All Fields] OR "vectorizing"[All Fields])) OR ("Malacology"[All Fields] AND ("survey s"[All Fields] OR "surveyed"[All Fields] OR "surveying"[All Fields] OR "surveys and questionnaires"[MeSH Terms] OR ("surveys"[All Fields] AND "questionnaires"[All Fields]) OR "surveys and questionnaires"[All Fields] OR "survey"[All Fields] OR "surveys"[All Fields])))

((("fasciola"[MeSH Terms] OR "fasciola"[All Fields]) AND ("intermediate"[All Fields] OR "intermediated"[All Fields] OR "intermediately"[All Fields] OR "intermediates"[All Fields]) AND "host"[All Fields]) OR (("intermediate"[All Fields] OR "intermediated"[All Fields] OR "intermediately"[All Fields] OR "intermediates"[All Fields]) AND "host"[All Fields] AND ("snail s"[All Fields] OR "snails"[MeSH Terms] OR "snails"[All Fields] OR "snail"[All Fields])) OR (("fresh water"[MeSH Terms] OR ("fresh"[All Fields] AND "water"[All Fields]) OR "fresh water"[All Fields] OR "freshwater"[All Fields] OR "freshwaters"[All Fields]) AND ("snail s"[All Fields] OR "snails"[MeSH Terms] OR "snails"[All Fields] OR "snail"[All Fields])) OR (("snail s"[All Fields] OR "snails"[MeSH Terms] OR "snails"[All Fields] OR "snail"[All Fields]) AND ("genetic vectors"[MeSH Terms] OR ("genetic"[All Fields] AND "vectors"[All Fields]) OR "genetic vectors"[All Fields] OR "vector"[All Fields] OR "vectors"[All Fields] OR "vector s"[All Fields] OR "vectored"[All Fields] OR "vectoring"[All Fields] OR "vectorization"[All Fields] OR "vectorize"[All Fields] OR "vectorized"[All Fields] OR "vectorizing"[All Fields])) OR ("Malacology"[All Fields] AND ("survey s"[All Fields] OR "surveyed"[All Fields] OR "surveying"[All Fields] OR "surveys and questionnaires"[MeSH Terms] OR ("surveys"[All Fields] AND "questionnaires"[All Fields]) OR "surveys and questionnaires"[All Fields] OR "survey"[All Fields] OR "surveys"[All Fields]))) AND ((("lymnaea"[MeSH Terms] OR "lymnaea"[All Fields] OR "lymnaeas"[All Fields]) AND ("columella"[All Fields] OR "columellae"[All Fields] OR "columellas"[All Fields])) OR (("lymnaea"[MeSH Terms] OR "lymnaea"[All Fields] OR "lymnaeas"[All Fields]) AND "natalensis"[All Fields]) OR (("lymnaea"[MeSH Terms] OR "lymnaea"[All Fields] OR "lymnaeas"[All Fields]) AND "truncatula"[All Fields]) OR ("Pseudosuccinea"[All Fields] AND ("columella"[All Fields] OR "columellae"[All Fields] OR "columellas"[All Fields])) OR (("plant roots"[MeSH Terms] OR ("plant"[All Fields] AND "roots"[All Fields]) OR "plant roots"[All Fields] OR "radix"[All Fields] OR "radixes"[All Fields]) AND "natalensis"[All Fields]) OR ("Galba"[All Fields] AND "truncatula"[All Fields])) AND ("fascioliasis"[MeSH Terms] OR "fascioliasis"[All Fields] OR ("fasciola hepatica"[MeSH Terms] OR ("fasciola"[All Fields] AND "hepatica"[All Fields]) OR "fasciola hepatica"[All Fields] OR ("liver"[All Fields] AND "fluke"[All Fields]) OR "liver fluke"[All Fields]) OR (("fasciola"[MeSH Terms] OR "fasciola"[All Fields]) AND "gigantica"[All Fields]) OR ("fasciola hepatica"[MeSH Terms] OR ("fasciola"[All Fields] AND "hepatica"[All Fields]) OR "fasciola hepatica"[All Fields]) OR ("fasciola"[MeSH Terms] OR "fasciola"[All Fields] OR ("fasciola"[All Fields] AND "sp"[All Fields]) OR "fasciola sp"[All Fields])) AND ((("algeria"[MeSH Terms] OR "algeria"[All Fields] OR ("egypt"[MeSH Terms] OR "egypt"[All Fields] OR "egypt s"[All Fields]) OR ("libya"[MeSH Terms] OR "libya"[All Fields]) OR "Morroco"[All Fields] OR ("sudan"[MeSH Terms] OR "sudan"[All Fields] OR "sudans"[All Fields] OR "sudan s"[All Fields]) OR ("tunisia"[MeSH Terms] OR "tunisia"[All Fields]) OR ("angola"[MeSH Terms] OR "angola"[All Fields] OR "angola s"[All Fields]) OR ("cameroon"[MeSH Terms] OR "cameroon"[All Fields] OR "cameroons"[All Fields] OR "cameroon s"[All Fields]) OR ("central african republic"[MeSH Terms] OR ("central"[All Fields] AND "african"[All Fields] AND "republic"[All Fields]) OR "central african republic"[All Fields]) OR ("chad"[MeSH Terms] OR "chad"[All Fields]) OR (("congo"[MeSH Terms] OR "congo"[All Fields]) AND ("republic"[All Fields] OR "republic s"[All Fields] OR "republics"[All Fields])) OR "Brazzaville"[All Fields] OR (("democrat"[All Fields] OR "democratic"[All Fields] OR "democratically"[All Fields] OR "democratization"[All Fields] OR "democratize"[All Fields] OR "democratized"[All Fields] OR "democratizing"[All Fields] OR "democrats"[All Fields]) AND

("republic"[All Fields] OR "republic s"[All Fields] OR "republics"[All Fields]) AND ("congo"[MeSH Terms] OR "congo"[All Fields])) OR ("equatorial guinea"[MeSH Terms] OR ("equatorial"[All Fields] AND "guinea"[All Fields]) OR "equatorial guinea"[All Fields]) OR ("gabon"[MeSH Terms] OR "gabon"[All Fields]) OR ("Sao"[All Fields] AND "Tome"[All Fields]) OR ("Sao"[All Fields] AND "Tome"[All Fields] AND ("principe"[All Fields] OR "principes"[All Fields])) OR ("botswana"[MeSH Terms] OR "botswana"[All Fields] OR "botswana s"[All Fields]) OR ("lesotho"[MeSH Terms] OR "lesotho"[All Fields]) OR ("namibia"[MeSH Terms] OR "namibia"[All Fields] OR "namibia s"[All Fields]) OR ("south africa"[MeSH Terms] OR ("south"[All Fields] AND "africa"[All Fields]) OR "south africa"[All Fields]) OR ("zimbabwe"[MeSH Terms] OR "zimbabwe"[All Fields] OR "zimbabwe s"[All Fields]) OR ("zambia"[MeSH Terms] OR "zambia"[All Fields] OR "zambia s"[All Fields]) OR ("mozambique"[MeSH Terms] OR "mozambique"[All Fields] OR "mozambique s"[All Fields]) OR ("mauritius"[MeSH Terms] OR "mauritius"[All Fields]) OR ("eswatini"[MeSH Terms] OR "eswatini"[All Fields] OR "swaziland"[All Fields]) OR ("madagascar"[MeSH Terms] OR "madagascar"[All Fields] OR "madagascar s"[All Fields]) OR ("mauritius"[MeSH Terms] OR "mauritius"[All Fields]) OR ("burundi"[MeSH Terms] OR "burundi"[All Fields]) OR ("comoros"[MeSH Terms] OR "comoros"[All Fields] OR "comoro"[All Fields]) OR ("djibouti"[MeSH Terms] OR "djibouti"[All Fields]) OR ("ethiopia"[MeSH Terms] OR "ethiopia"[All Fields] OR "ethiopia s"[All Fields]) OR ("eritrea"[MeSH Terms] OR "eritrea"[All Fields]) OR ("kenya"[MeSH Terms] OR "kenya"[All Fields] OR "kenya s"[All Fields]) OR ("rwanda"[MeSH Terms] OR "rwanda"[All Fields] OR "rwanda s"[All Fields]) OR ("seychelles"[MeSH Terms] OR "seychelles"[All Fields]) OR ("somalia"[MeSH Terms] OR "somalia"[All Fields] OR "somalia s"[All Fields]) OR ("tanzania"[MeSH Terms] OR "tanzania"[All Fields] OR "tanzania s"[All Fields]) OR ("uganda"[MeSH Terms] OR "uganda"[All Fields] OR "uganda s"[All Fields]) OR ("benin"[MeSH Terms] OR "benin"[All Fields] OR "benin s"[All Fields])) AND ("burkina faso"[MeSH Terms] OR ("burkina"[All Fields] AND "faso"[All Fields]) OR "burkina faso"[All Fields])) OR ("cabo verde"[MeSH Terms] OR ("cabo"[All Fields] AND "verde"[All Fields]) OR "cabo verde"[All Fields] OR ("cape"[All Fields] AND "verde"[All Fields]) OR "cape verde"[All Fields]) OR ("cote d ivoire"[MeSH Terms] OR ("cote"[All Fields] AND "d ivoire"[All Fields]) OR "cote d ivoire"[All Fields]) OR ("gambia"[MeSH Terms] OR "gambia"[All Fields] OR "gambia s"[All Fields]) OR ("ghana"[MeSH Terms] OR "ghana"[All Fields] OR "ghana s"[All Fields]) OR ("guinea"[MeSH Terms] OR "guinea"[All Fields] OR "guinea s"[All Fields] OR "guineas"[All Fields]) OR ("guinea bissau"[MeSH Terms] OR "guinea bissau"[All Fields] OR ("guinea"[All Fields] AND "bissau"[All Fields]) OR "guinea bissau"[All Fields]) OR ("liberia"[MeSH Terms] OR "liberia"[All Fields] OR "liberia s"[All Fields]) OR ("mali"[MeSH Terms] OR "mali"[All Fields]) OR ("mauritania"[MeSH Terms] OR "mauritania"[All Fields]) OR ("niger"[MeSH Terms] OR "niger"[All Fields]) OR ("nigeria"[MeSH Terms] OR "nigeria"[All Fields] OR "nigeria s"[All Fields]) OR (("sierra"[All Fields] OR "sierras"[All Fields]) AND "Eone"[All Fields]) OR ("sierra leone"[MeSH Terms] OR ("sierra"[All Fields] AND "leone"[All Fields]) OR "sierra leone"[All Fields]) OR ("togo"[MeSH Terms] OR "togo"[All Fields]))

((("fasciola"[MeSH Terms] OR "fasciola"[All Fields]) AND ("intermediate"[All Fields] OR "intermediated"[All Fields] OR "intermediately"[All Fields] OR "intermediates"[All Fields]) AND "host"[All Fields]) OR (("intermediate"[All Fields] OR "intermediated"[All Fields] OR "intermediately"[All Fields] OR "intermediates"[All Fields]) AND "host"[All Fields] AND ("snail s"[All Fields] OR "snails"[MeSH Terms] OR "snails"[All Fields] OR "snail"[All Fields])) OR (("fresh water"[MeSH Terms] OR ("fresh"[All Fields] AND "water"[All Fields]) OR "fresh water"[All Fields] OR "freshwater"[All Fields] OR "freshwaters"[All Fields]) AND ("snail s"[All Fields] OR "snails"[MeSH Terms] OR "snails"[All Fields] OR "snail"[All Fields])) OR (("snail s"[All Fields] OR "snails"[MeSH Terms] OR "snails"[All Fields] OR "snail"[All Fields]) AND ("genetic vectors"[MeSH Terms] OR ("genetic"[All Fields] AND "vectors"[All Fields]) OR "genetic vectors"[All Fields] OR "vector"[All Fields] OR "vectors"[All Fields] OR "vector s"[All Fields] OR "vectored"[All Fields] OR "vectoring"[All Fields] OR

"vectorization"[All Fields] OR "vectorize"[All Fields] OR "vectorized"[All Fields] OR "vectorizing"[All Fields])) OR ("Malacology"[All Fields] AND ("survey s"[All Fields] OR "surveyed"[All Fields] OR "surveying"[All Fields] OR "surveys and questionnaires"[MeSH Terms] OR ("surveys"[All Fields] AND "questionnaires"[All Fields]) OR "surveys and questionnaires"[All Fields] OR "survey"[All Fields] OR "surveys"[All Fields]))) AND ((("lymnaea"[MeSH Terms] OR "lymnaea"[All Fields] OR "lymnaeas"[All Fields]) AND ("columella"[All Fields] OR "columellae"[All Fields] OR "columellas"[All Fields])) OR (("lymnaea"[MeSH Terms] OR "lymnaea"[All Fields] OR "lymnaeas"[All Fields]) AND "natalensis"[All Fields]) OR (("lymnaea"[MeSH Terms] OR "lymnaea"[All Fields] OR "lymnaeas"[All Fields]) AND "truncatula"[All Fields]) OR ("Pseudosuccinea"[All Fields] AND ("columella"[All Fields] OR "columellae"[All Fields] OR "columellas"[All Fields])) OR (("plant roots"[MeSH Terms] OR ("plant"[All Fields] AND "roots"[All Fields]) OR "plant roots"[All Fields] OR "radix"[All Fields] OR "radixes"[All Fields]) AND "natalensis"[All Fields]) OR ("Galba"[All Fields] AND "truncatula"[All Fields])) AND ("fascioliasis"[MeSH Terms] OR "fascioliasis"[All Fields] OR ("fasciola hepatica"[MeSH Terms] OR ("fasciola"[All Fields] AND "hepatica"[All Fields]) OR "fasciola hepatica"[All Fields] OR ("liver"[All Fields] AND "fluke"[All Fields]) OR "liver fluke"[All Fields]) OR (("fasciola"[MeSH Terms] OR "fasciola"[All Fields]) AND "gigantica"[All Fields]) OR ("fasciola hepatica"[MeSH Terms] OR ("fasciola"[All Fields] AND "hepatica"[All Fields]) OR "fasciola hepatica"[All Fields]) OR ("fasciola"[MeSH Terms] OR "fasciola"[All Fields] OR ("fasciola"[All Fields] AND "sp"[All Fields]) OR "fasciola sp"[All Fields]))

("infect"[All Fields] OR "infectability"[All Fields] OR "infectable"[All Fields] OR "infectant"[All Fields] OR "infectants"[All Fields] OR "infected"[All Fields] OR "infecteds"[All Fields] OR "infectibility"[All Fields] OR "infectible"[All Fields] OR "infecting"[All Fields] OR "infection s"[All Fields] OR "infections"[MeSH Terms] OR "infections"[All Fields] OR "infection"[All Fields] OR "infective"[All Fields] OR "infectiveness"[All Fields] OR "infectives"[All Fields] OR "infectivities"[All Fields] OR "infects"[All Fields] OR "pathogenicity"[MeSH Subheading] OR "pathogenicity"[All Fields] OR "infectivity"[All Fields] OR (("infect"[All Fields] OR "infectability"[All Fields] OR "infectable"[All Fields] OR "infectant"[All Fields] OR "infectants"[All Fields] OR "infected"[All Fields] OR "infecteds"[All Fields] OR "infectibility"[All Fields] OR "infectible"[All Fields] OR "infecting"[All Fields] OR "infection s"[All Fields] OR "infections"[MeSH Terms] OR "infections"[All Fields] OR "infection"[All Fields] OR "infective"[All Fields] OR "infectiveness"[All Fields] OR "infectives"[All Fields] OR "infectivities"[All Fields] OR "infects"[All Fields] OR "pathogenicity"[MeSH Subheading] OR "pathogenicity"[All Fields] OR "infectivity"[All Fields]) AND ("j rehabil assist technol eng"[Journal] OR "rate"[All Fields])) OR ("intense"[All Fields] OR "intensely"[All Fields] OR "intensities"[All Fields] OR "intensity"[All Fields] OR "intensively"[All Fields]) OR ("epidemiology"[MeSH Subheading] OR "epidemiology"[All Fields] OR "prevalence"[All Fields] OR "prevalence"[MeSH Terms] OR "prevalance"[All Fields] OR "prevalences"[All Fields] OR "prevalence s"[All Fields] OR "prevalent"[All Fields] OR "prevalently"[All Fields] OR "prevalents"[All Fields]) OR ("epidemiology"[MeSH Subheading] OR "epidemiology"[All Fields] OR "incidence"[All Fields] OR "incidence"[MeSH Terms] OR "incidences"[All Fields] OR "incident"[All Fields] OR "incidents"[All Fields])) AND (((("fasciola"[MeSH Terms] OR "fasciola"[All Fields]) AND ("intermediate"[All Fields] OR "intermediated"[All Fields] OR "intermediately"[All Fields] OR "intermediates"[All Fields]) AND "host"[All Fields]) OR (("intermediate"[All Fields] OR "intermediated"[All Fields] OR "intermediately"[All Fields] OR "intermediates"[All Fields]) AND "host"[All Fields] AND ("snail s"[All Fields] OR "snails"[MeSH Terms] OR "snails"[All Fields] OR "snail"[All Fields])) OR (("fresh water"[MeSH Terms] OR ("fresh"[All Fields] AND "water"[All Fields]) OR "fresh water"[All Fields] OR "freshwater"[All Fields] OR "freshwaters"[All Fields]) AND ("snail s"[All Fields] OR "snails"[MeSH Terms] OR "snails"[All Fields] OR "snail"[All Fields])) OR (("snail s"[All Fields] OR "snails"[MeSH Terms] OR "snails"[All Fields] OR "snail"[All Fields]) AND ("genetic

vectors"[MeSH Terms] OR ("genetic"[All Fields] AND "vectors"[All Fields]) OR "genetic vectors"[All Fields] OR "vector"[All Fields] OR "vectors"[All Fields] OR "vector s"[All Fields] OR "vectored"[All Fields] OR "vectoring"[All Fields] OR "vectorization"[All Fields] OR "vectorize"[All Fields] OR "vectorized"[All Fields] OR "vectorizing"[All Fields])) OR ("Malacology"[All Fields] AND ("survey s"[All Fields] OR "surveyed"[All Fields] OR "surveying"[All Fields] OR "surveys and questionnaires"[MeSH Terms] OR ("surveys"[All Fields] AND "questionnaires"[All Fields]) OR "surveys and questionnaires"[All Fields] OR "survey"[All Fields] OR "surveys"[All Fields]))) AND ((("lymnaea"[MeSH Terms] OR "lymnaea"[All Fields] OR "lymnaeas"[All Fields]) AND ("columella"[All Fields] OR "columellae"[All Fields] OR "columellas"[All Fields])) OR (("lymnaea"[MeSH Terms] OR "lymnaea"[All Fields] OR "lymnaeas"[All Fields]) AND "natalensis"[All Fields]) OR (("lymnaea"[MeSH Terms] OR "lymnaea"[All Fields] OR "lymnaeas"[All Fields]) AND "truncatula"[All Fields]) OR ("Pseudosuccinea"[All Fields] AND ("columella"[All Fields] OR "columellae"[All Fields] OR "columellas"[All Fields])) OR (("plant roots"[MeSH Terms] OR ("plant"[All Fields] AND "roots"[All Fields]) OR "plant roots"[All Fields] OR "radix"[All Fields] OR "radixes"[All Fields]) AND "natalensis"[All Fields]) OR ("Galba"[All Fields] AND "truncatula"[All Fields]))) AND ((("algeria"[MeSH Terms] OR "algeria"[All Fields] OR ("egypt"[MeSH Terms] OR "egypt"[All Fields] OR "egypt s"[All Fields]) OR ("libya"[MeSH Terms] OR "libya"[All Fields]) OR "Morroco"[All Fields] OR ("sudan"[MeSH Terms] OR "sudan"[All Fields] OR "sudans"[All Fields] OR "sudan s"[All Fields]) OR ("tunisia"[MeSH Terms] OR "tunisia"[All Fields]) OR ("angola"[MeSH Terms] OR "angola"[All Fields] OR "angola s"[All Fields]) OR ("cameroon"[MeSH Terms] OR "cameroon"[All Fields] OR "cameroons"[All Fields] OR "cameroon s"[All Fields]) OR ("central african republic"[MeSH Terms] OR ("central"[All Fields] AND "african"[All Fields] AND "republic"[All Fields]) OR "central african republic"[All Fields]) OR ("chad"[MeSH Terms] OR "chad"[All Fields]) OR (("congo"[MeSH Terms] OR "congo"[All Fields]) AND ("republic"[All Fields] OR "republic s"[All Fields] OR "republics"[All Fields])) OR "Brazzaville"[All Fields] OR (("democrat"[All Fields] OR "democratic"[All Fields] OR "democratically"[All Fields] OR "democratization"[All Fields] OR "democratize"[All Fields] OR "democratized"[All Fields] OR "democratizing"[All Fields] OR "democrats"[All Fields]) AND ("republic"[All Fields] OR "republic s"[All Fields] OR "republics"[All Fields]) AND ("congo"[MeSH Terms] OR "congo"[All Fields])) OR ("equatorial guinea"[MeSH Terms] OR ("equatorial"[All Fields] AND "guinea"[All Fields]) OR "equatorial guinea"[All Fields]) OR ("gabon"[MeSH Terms] OR "gabon"[All Fields]) OR ("Sao"[All Fields] AND "Tome"[All Fields]) OR ("Sao"[All Fields] AND "Tome"[All Fields] AND ("principe"[All Fields] OR "principes"[All Fields])) OR ("botswana"[MeSH Terms] OR "botswana"[All Fields] OR "botswana s"[All Fields]) OR ("lesotho"[MeSH Terms] OR "lesotho"[All Fields]) OR ("namibia"[MeSH Terms] OR "namibia"[All Fields] OR "namibia s"[All Fields]) OR ("south africa"[MeSH Terms] OR ("south"[All Fields] AND "africa"[All Fields]) OR "south africa"[All Fields]) OR ("zimbabwe"[MeSH Terms] OR "zimbabwe"[All Fields] OR "zimbabwe s"[All Fields]) OR ("zambia"[MeSH Terms] OR "zambia"[All Fields] OR "zambia s"[All Fields]) OR ("mozambique"[MeSH Terms] OR "mozambique"[All Fields] OR "mozambique s"[All Fields]) OR ("mauritius"[MeSH Terms] OR "mauritius"[All Fields]) OR ("eswatini"[MeSH Terms] OR "eswatini"[All Fields] OR "swaziland"[All Fields]) OR ("madagascar"[MeSH Terms] OR "madagascar"[All Fields] OR "madagascar s"[All Fields]) OR ("mauritius"[MeSH Terms] OR "mauritius"[All Fields]) OR ("burundi"[MeSH Terms] OR "burundi"[All Fields]) OR ("comoros"[MeSH Terms] OR "comoros"[All Fields] OR "comoro"[All Fields]) OR ("djibouti"[MeSH Terms] OR "djibouti"[All Fields]) OR ("ethiopia"[MeSH Terms] OR "ethiopia"[All Fields] OR "ethiopia s"[All Fields]) OR ("eritrea"[MeSH Terms] OR "eritrea"[All Fields]) OR ("kenya"[MeSH Terms] OR "kenya"[All Fields] OR "kenya s"[All Fields]) OR ("rwanda"[MeSH Terms] OR "rwanda"[All Fields] OR "rwanda s"[All Fields]) OR ("seychelles"[MeSH Terms] OR "seychelles"[All Fields]) OR ("somalia"[MeSH Terms] OR "somalia"[All Fields] OR "somalia s"[All Fields]) OR ("tanzania"[MeSH Terms] OR "tanzania"[All Fields] OR "tanzania s"[All Fields]) OR ("uganda"[MeSH Terms] OR "uganda"[All Fields] OR "uganda

s"[All Fields]) OR ("benin"[MeSH Terms] OR "benin"[All Fields] OR "benin s"[All Fields])) AND ("burkina faso"[MeSH Terms] OR ("burkina"[All Fields] AND "faso"[All Fields]) OR "burkina faso"[All Fields])) OR ("cabo verde"[MeSH Terms] OR ("cabo"[All Fields] AND "verde"[All Fields]) OR "cabo verde"[All Fields] OR ("cape"[All Fields] AND "verde"[All Fields]) OR "cape verde"[All Fields]) OR ("cote d ivoire"[MeSH Terms] OR ("cote"[All Fields] AND "d ivoire"[All Fields]) OR "cote d ivoire"[All Fields]) OR ("gambia"[MeSH Terms] OR "gambia"[All Fields] OR "gambia s"[All Fields]) OR ("ghana"[MeSH Terms] OR "ghana"[All Fields] OR "ghana s"[All Fields]) OR ("guinea"[MeSH Terms] OR "guinea"[All Fields] OR "guinea s"[All Fields] OR "guineas"[All Fields]) OR ("guinea bissau"[MeSH Terms] OR "guinea bissau"[All Fields] OR ("guinea"[All Fields] AND "bissau"[All Fields]) OR "guinea bissau"[All Fields]) OR ("liberia"[MeSH Terms] OR "liberia"[All Fields] OR "liberia s"[All Fields]) OR ("mali"[MeSH Terms] OR "mali"[All Fields]) OR ("mauritania"[MeSH Terms] OR "mauritania"[All Fields]) OR ("niger"[MeSH Terms] OR "niger"[All Fields]) OR ("nigeria"[MeSH Terms] OR "nigeria"[All Fields] OR "nigeria s"[All Fields]) OR (("sierra"[All Fields] OR "sierras"[All Fields]) AND "Eone"[All Fields]) OR ("sierra leone"[MeSH Terms] OR ("sierra"[All Fields] AND "leone"[All Fields]) OR "sierra leone"[All Fields]) OR ("togo"[MeSH Terms] OR "togo"[All Fields]))

(("infect"[All Fields] OR "infectability"[All Fields] OR "infectable"[All Fields] OR "infectant"[All Fields] OR "infectants"[All Fields] OR "infected"[All Fields] OR "infecteds"[All Fields] OR "infectibility"[All Fields] OR "infectible"[All Fields] OR "infecting"[All Fields] OR "infection s"[All Fields] OR "infections"[MeSH Terms] OR "infections"[All Fields] OR "infection"[All Fields] OR "infective"[All Fields] OR "infectiveness"[All Fields] OR "infectives"[All Fields] OR "infectivities"[All Fields] OR "infects"[All Fields] OR "pathogenicity"[MeSH Subheading] OR "pathogenicity"[All Fields] OR "infectivity"[All Fields] OR (("infect"[All Fields] OR "infectability"[All Fields] OR "infectable"[All Fields] OR "infectant"[All Fields] OR "infectants"[All Fields] OR "infected"[All Fields] OR "infecteds"[All Fields] OR "infectibility"[All Fields] OR "infectible"[All Fields] OR "infecting"[All Fields] OR "infection s"[All Fields] OR "infections"[MeSH Terms] OR "infections"[All Fields] OR "infection"[All Fields] OR "infective"[All Fields] OR "infectiveness"[All Fields] OR "infectives"[All Fields] OR "infectivities"[All Fields] OR "infects"[All Fields] OR "pathogenicity"[MeSH Subheading] OR "pathogenicity"[All Fields] OR "infectivity"[All Fields]) AND ("j rehabil assist technol eng"[Journal] OR "rate"[All Fields])) OR ("intense"[All Fields] OR "intensely"[All Fields] OR "intensities"[All Fields] OR "intensity"[All Fields] OR "intensively"[All Fields]) OR ("epidemiology"[MeSH Subheading] OR "epidemiology"[All Fields] OR "prevalence"[All Fields] OR "prevalence"[MeSH Terms] OR "prevalance"[All Fields] OR "prevalences"[All Fields] OR "prevalence s"[All Fields] OR "prevalent"[All Fields] OR "prevalently"[All Fields] OR "prevalents"[All Fields]) OR ("epidemiology"[MeSH Subheading] OR "epidemiology"[All Fields] OR "incidence"[All Fields] OR "incidence"[MeSH Terms] OR "incidences"[All Fields] OR "incident"[All Fields] OR "incidents"[All Fields])) AND (((("fasciola"[MeSH Terms] OR "fasciola"[All Fields]) AND ("intermediate"[All Fields] OR "intermediated"[All Fields] OR "intermediately"[All Fields] OR "intermediates"[All Fields]) AND "host"[All Fields]) OR (("intermediate"[All Fields] OR "intermediated"[All Fields] OR "intermediately"[All Fields] OR "intermediates"[All Fields]) AND "host"[All Fields] AND ("snail s"[All Fields] OR "snails"[MeSH Terms] OR "snails"[All Fields] OR "snail"[All Fields])) OR (("fresh water"[MeSH Terms] OR ("fresh"[All Fields] AND "water"[All Fields]) OR "fresh water"[All Fields] OR "freshwater"[All Fields] OR "freshwaters"[All Fields]) AND ("snail s"[All Fields] OR "snails"[MeSH Terms] OR "snails"[All Fields] OR "snail"[All Fields])) OR (("snail s"[All Fields] OR "snails"[MeSH Terms] OR "snails"[All Fields] OR "snail"[All Fields]) AND ("genetic vectors"[MeSH Terms] OR ("genetic"[All Fields] AND "vectors"[All Fields]) OR "genetic vectors"[All Fields] OR "vector"[All Fields] OR "vectors"[All Fields] OR "vector s"[All Fields] OR "vectored"[All Fields] OR "vectoring"[All Fields] OR "vectorization"[All Fields] OR "vectorize"[All Fields] OR "vectorized"[All Fields] OR "vectorizing"[All Fields])) OR ("Malacology"[All Fields] AND ("survey s"[All

Fields] OR "surveyed"[All Fields] OR "surveying"[All Fields] OR "surveys and questionnaires"[MeSH Terms] OR ("surveys"[All Fields] AND "questionnaires"[All Fields]) OR "surveys and questionnaires"[All Fields] OR "survey"[All Fields] OR "surveys"[All Fields]))) AND ((("lymnaea"[MeSH Terms] OR "lymnaea"[All Fields] OR "lymnaeas"[All Fields]) AND ("columella"[All Fields] OR "columellae"[All Fields] OR "columellas"[All Fields])) OR (("lymnaea"[MeSH Terms] OR "lymnaea"[All Fields] OR "lymnaeas"[All Fields]) AND "natalensis"[All Fields]) OR (("lymnaea"[MeSH Terms] OR "lymnaea"[All Fields] OR "lymnaeas"[All Fields]) AND "truncatula"[All Fields]) OR ("Pseudosuccinea"[All Fields] AND ("columella"[All Fields] OR "columellae"[All Fields] OR "columellas"[All Fields])) OR (("plant roots"[MeSH Terms] OR ("plant"[All Fields] AND "roots"[All Fields]) OR "plant roots"[All Fields] OR "radix"[All Fields] OR "radixes"[All Fields]) AND "natalensis"[All Fields]) OR ("Galba"[All Fields] AND "truncatula"[All Fields])))) OR ("fascioliasis"[MeSH Terms] OR "fascioliasis"[All Fields] OR ("fasciola hepatica"[MeSH Terms] OR ("fasciola"[All Fields] AND "hepatica"[All Fields]) OR "fasciola hepatica"[All Fields] OR ("liver"[All Fields] AND "fluke"[All Fields]) OR "liver fluke"[All Fields]) OR (("fasciola"[MeSH Terms] OR "fasciola"[All Fields]) AND "gigantica"[All Fields]) OR ("fasciola hepatica"[MeSH Terms] OR ("fasciola"[All Fields] AND "hepatica"[All Fields]) OR "fasciola hepatica"[All Fields]) OR ("fasciola"[MeSH Terms] OR "fasciola"[All Fields] OR ("fasciola"[All Fields] AND "sp"[All Fields]) OR "fasciola sp"[All Fields]))

("infect"[All Fields] OR "infectability"[All Fields] OR "infectable"[All Fields] OR "infectant"[All Fields] OR "infectants"[All Fields] OR "infected"[All Fields] OR "infecteds"[All Fields] OR "infectibility"[All Fields] OR "infectible"[All Fields] OR "infecting"[All Fields] OR "infection s"[All Fields] OR "infections"[MeSH Terms] OR "infections"[All Fields] OR "infection"[All Fields] OR "infective"[All Fields] OR "infectiveness"[All Fields] OR "infectives"[All Fields] OR "infectivities"[All Fields] OR "infects"[All Fields] OR "pathogenicity"[MeSH Subheading] OR "pathogenicity"[All Fields] OR "infectivity"[All Fields] OR (("infect"[All Fields] OR "infectability"[All Fields] OR "infectable"[All Fields] OR "infectant"[All Fields] OR "infectants"[All Fields] OR "infected"[All Fields] OR "infecteds"[All Fields] OR "infectibility"[All Fields] OR "infectible"[All Fields] OR "infecting"[All Fields] OR "infection s"[All Fields] OR "infections"[MeSH Terms] OR "infections"[All Fields] OR "infection"[All Fields] OR "infective"[All Fields] OR "infectiveness"[All Fields] OR "infectives"[All Fields] OR "infectivities"[All Fields] OR "infects"[All Fields] OR "pathogenicity"[MeSH Subheading] OR "pathogenicity"[All Fields] OR "infectivity"[All Fields]) AND ("j rehabil assist technol eng"[Journal] OR "rate"[All Fields])) OR ("intense"[All Fields] OR "intensely"[All Fields] OR "intensities"[All Fields] OR "intensity"[All Fields] OR "intensively"[All Fields]) OR ("epidemiology"[MeSH Subheading] OR "epidemiology"[All Fields] OR "prevalence"[All Fields] OR "prevalence"[MeSH Terms] OR "prevalance"[All Fields] OR "prevalences"[All Fields] OR "prevalence s"[All Fields] OR "prevalent"[All Fields] OR "prevalently"[All Fields] OR "prevalents"[All Fields]) OR ("epidemiology"[MeSH Subheading] OR "epidemiology"[All Fields] OR "incidence"[All Fields] OR "incidence"[MeSH Terms] OR "incidences"[All Fields] OR "incident"[All Fields] OR "incidents"[All Fields])) AND (((("fasciola"[MeSH Terms] OR "fasciola"[All Fields]) AND ("intermediate"[All Fields] OR "intermediated"[All Fields] OR "intermediately"[All Fields] OR "intermediates"[All Fields]) AND "host"[All Fields]) OR (("intermediate"[All Fields] OR "intermediated"[All Fields] OR "intermediately"[All Fields] OR "intermediates"[All Fields]) AND "host"[All Fields] AND ("snail s"[All Fields] OR "snails"[MeSH Terms] OR "snails"[All Fields] OR "snail"[All Fields])) OR (("fresh water"[MeSH Terms] OR ("fresh"[All Fields] AND "water"[All Fields]) OR "fresh water"[All Fields] OR "freshwater"[All Fields] OR "freshwaters"[All Fields]) AND ("snail s"[All Fields] OR "snails"[MeSH Terms] OR "snails"[All Fields] OR "snail"[All Fields])) OR (("snail s"[All Fields] OR "snails"[MeSH Terms] OR "snails"[All Fields] OR "snail"[All Fields]) AND ("genetic vectors"[MeSH Terms] OR ("genetic"[All Fields] AND "vectors"[All Fields]) OR "genetic vectors"[All Fields] OR "vector"[All Fields] OR "vectors"[All Fields] OR "vector s"[All Fields] OR "vectored"[All

Fields] OR "vectoring"[All Fields] OR "vectorization"[All Fields] OR "vectorize"[All Fields] OR "vectorized"[All Fields] OR "vectorizing"[All Fields])) OR ("Malacology"[All Fields] AND ("survey s"[All Fields] OR "surveyed"[All Fields] OR "surveying"[All Fields] OR "surveys and questionnaires"[MeSH Terms] OR ("surveys"[All Fields] AND "questionnaires"[All Fields]) OR "surveys and questionnaires"[All Fields] OR "survey"[All Fields] OR "surveys"[All Fields]))) AND ((("lymnaea"[MeSH Terms] OR "lymnaea"[All Fields] OR "lymnaeas"[All Fields]) AND ("columella"[All Fields] OR "columellae"[All Fields] OR "columellas"[All Fields])) OR (("lymnaea"[MeSH Terms] OR "lymnaea"[All Fields] OR "lymnaeas"[All Fields]) AND "natalensis"[All Fields]) OR (("lymnaea"[MeSH Terms] OR "lymnaea"[All Fields] OR "lymnaeas"[All Fields]) AND "truncatula"[All Fields]) OR ("Pseudosuccinea"[All Fields] AND ("columella"[All Fields] OR "columellae"[All Fields] OR "columellas"[All Fields])) OR (("plant roots"[MeSH Terms] OR ("plant"[All Fields] AND "roots"[All Fields]) OR "plant roots"[All Fields] OR "radix"[All Fields] OR "radixes"[All Fields]) AND "natalensis"[All Fields]) OR ("Galba"[All Fields] AND "truncatula"[All Fields])))

((("fasciola"[MeSH Terms] OR "fasciola"[All Fields]) AND ("intermediate"[All Fields] OR "intermediated"[All Fields] OR "intermediately"[All Fields] OR "intermediates"[All Fields]) AND "host"[All Fields]) OR (("intermediate"[All Fields] OR "intermediated"[All Fields] OR "intermediately"[All Fields] OR "intermediates"[All Fields]) AND "host"[All Fields] AND ("snail s"[All Fields] OR "snails"[MeSH Terms] OR "snails"[All Fields] OR "snail"[All Fields])) OR (("fresh water"[MeSH Terms] OR ("fresh"[All Fields] AND "water"[All Fields]) OR "fresh water"[All Fields] OR "freshwater"[All Fields] OR "freshwaters"[All Fields]) AND ("snail s"[All Fields] OR "snails"[MeSH Terms] OR "snails"[All Fields] OR "snail"[All Fields])) OR (("snail s"[All Fields] OR "snails"[MeSH Terms] OR "snails"[All Fields] OR "snail"[All Fields]) AND ("genetic vectors"[MeSH Terms] OR ("genetic"[All Fields] AND "vectors"[All Fields]) OR "genetic vectors"[All Fields] OR "vector"[All Fields] OR "vectors"[All Fields] OR "vector s"[All Fields] OR "vectored"[All Fields] OR "vectoring"[All Fields] OR "vectorization"[All Fields] OR "vectorize"[All Fields] OR "vectorized"[All Fields] OR "vectorizing"[All Fields])) OR ("Malacology"[All Fields] AND ("survey s"[All Fields] OR "surveyed"[All Fields] OR "surveying"[All Fields] OR "surveys and questionnaires"[MeSH Terms] OR ("surveys"[All Fields] AND "questionnaires"[All Fields]) OR "surveys and questionnaires"[All Fields] OR "survey"[All Fields] OR "surveys"[All Fields]))) AND ((("lymnaea"[MeSH Terms] OR "lymnaea"[All Fields] OR "lymnaeas"[All Fields]) AND ("columella"[All Fields] OR "columellae"[All Fields] OR "columellas"[All Fields])) OR (("lymnaea"[MeSH Terms] OR "lymnaea"[All Fields] OR "lymnaeas"[All Fields]) AND "natalensis"[All Fields]) OR (("lymnaea"[MeSH Terms] OR "lymnaea"[All Fields] OR "lymnaeas"[All Fields]) AND "truncatula"[All Fields]) OR ("Pseudosuccinea"[All Fields] AND ("columella"[All Fields] OR "columellae"[All Fields] OR "columellas"[All Fields])) OR (("plant roots"[MeSH Terms] OR ("plant"[All Fields] AND "roots"[All Fields]) OR "plant roots"[All Fields] OR "radix"[All Fields] OR "radixes"[All Fields]) AND "natalensis"[All Fields]) OR ("Galba"[All Fields] AND "truncatula"[All Fields]))

(("algeria"[MeSH Terms] OR "algeria"[All Fields] OR ("egypt"[MeSH Terms] OR "egypt"[All Fields] OR "egypt s"[All Fields]) OR ("libya"[MeSH Terms] OR "libya"[All Fields]) OR "Morroco"[All Fields] OR ("sudan"[MeSH Terms] OR "sudan"[All Fields] OR "sudans"[All Fields] OR "sudan s"[All Fields]) OR ("tunisia"[MeSH Terms] OR "tunisia"[All Fields]) OR ("angola"[MeSH Terms] OR "angola"[All Fields] OR "angola s"[All Fields]) OR ("cameroon"[MeSH Terms] OR "cameroon"[All Fields] OR "cameroons"[All Fields] OR "cameroon s"[All Fields]) OR ("central african republic"[MeSH Terms] OR ("central"[All Fields] AND "african"[All Fields] AND "republic"[All Fields]) OR "central african republic"[All Fields]) OR ("chad"[MeSH Terms] OR "chad"[All Fields]) OR (("congo"[MeSH Terms] OR "congo"[All Fields]) AND ("republic"[All Fields] OR "republic s"[All Fields] OR "republics"[All Fields])) OR "Brazzaville"[All Fields] OR (("democrat"[All Fields] OR "democratic"[All Fields] OR "democratically"[All Fields] OR "democratization"[All Fields] OR "democratize"[All Fields] OR

"democratized"[All Fields] OR "democratizing"[All Fields] OR "democrats"[All Fields]) AND ("republic"[All Fields] OR "republic s"[All Fields] OR "republics"[All Fields]) AND ("congo"[MeSH Terms] OR "congo"[All Fields])) OR ("equatorial guinea"[MeSH Terms] OR ("equatorial"[All Fields] AND "guinea"[All Fields]) OR "equatorial guinea"[All Fields]) OR ("gabon"[MeSH Terms] OR "gabon"[All Fields]) OR ("Sao"[All Fields] AND "Tome"[All Fields]) OR ("Sao"[All Fields] AND "Tome"[All Fields] AND ("principe"[All Fields] OR "principes"[All Fields])) OR ("botswana"[MeSH Terms] OR "botswana"[All Fields] OR "botswana s"[All Fields]) OR ("lesotho"[MeSH Terms] OR "lesotho"[All Fields]) OR ("namibia"[MeSH Terms] OR "namibia"[All Fields] OR "namibia s"[All Fields]) OR ("south africa"[MeSH Terms] OR ("south"[All Fields] AND "africa"[All Fields]) OR "south africa"[All Fields]) OR ("zimbabwe"[MeSH Terms] OR "zimbabwe"[All Fields] OR "zimbabwe s"[All Fields]) OR ("zambia"[MeSH Terms] OR "zambia"[All Fields] OR "zambia s"[All Fields]) OR ("mozambique"[MeSH Terms] OR "mozambique"[All Fields] OR "mozambique s"[All Fields]) OR ("mauritius"[MeSH Terms] OR "mauritius"[All Fields]) OR ("eswatini"[MeSH Terms] OR "eswatini"[All Fields] OR "swaziland"[All Fields]) OR ("madagascar"[MeSH Terms] OR "madagascar"[All Fields] OR "madagascar s"[All Fields]) OR ("mauritius"[MeSH Terms] OR "mauritius"[All Fields]) OR ("burundi"[MeSH Terms] OR "burundi"[All Fields]) OR ("comoros"[MeSH Terms] OR "comoros"[All Fields] OR "comoro"[All Fields]) OR ("djibouti"[MeSH Terms] OR "djibouti"[All Fields]) OR ("ethiopia"[MeSH Terms] OR "ethiopia"[All Fields] OR "ethiopia s"[All Fields]) OR ("eritrea"[MeSH Terms] OR "eritrea"[All Fields]) OR ("kenya"[MeSH Terms] OR "kenya"[All Fields] OR "kenya s"[All Fields]) OR ("rwanda"[MeSH Terms] OR "rwanda"[All Fields] OR "rwanda s"[All Fields]) OR ("seychelles"[MeSH Terms] OR "seychelles"[All Fields]) OR ("somalia"[MeSH Terms] OR "somalia"[All Fields] OR "somalia s"[All Fields]) OR ("tanzania"[MeSH Terms] OR "tanzania"[All Fields] OR "tanzania s"[All Fields]) OR ("uganda"[MeSH Terms] OR "uganda"[All Fields] OR "uganda s"[All Fields]) OR ("benin"[MeSH Terms] OR "benin"[All Fields] OR "benin s"[All Fields])) AND ("burkina faso"[MeSH Terms] OR ("burkina"[All Fields] AND "faso"[All Fields]) OR "burkina faso"[All Fields])) OR ("cabo verde"[MeSH Terms] OR ("cabo"[All Fields] AND "verde"[All Fields]) OR "cabo verde"[All Fields] OR ("cape"[All Fields] AND "verde"[All Fields]) OR "cape verde"[All Fields]) OR ("cote d ivoire"[MeSH Terms] OR ("cote"[All Fields] AND "d ivoire"[All Fields]) OR "cote d ivoire"[All Fields]) OR ("gambia"[MeSH Terms] OR "gambia"[All Fields] OR "gambia s"[All Fields]) OR ("ghana"[MeSH Terms] OR "ghana"[All Fields] OR "ghana s"[All Fields]) OR ("guinea"[MeSH Terms] OR "guinea"[All Fields] OR "guinea s"[All Fields] OR "guineas"[All Fields]) OR ("guinea bissau"[MeSH Terms] OR "guinea bissau"[All Fields] OR ("guinea"[All Fields] AND "bissau"[All Fields]) OR "guinea bissau"[All Fields]) OR ("liberia"[MeSH Terms] OR "liberia"[All Fields] OR "liberia s"[All Fields]) OR ("mali"[MeSH Terms] OR "mali"[All Fields]) OR ("mauritania"[MeSH Terms] OR "mauritania"[All Fields]) OR ("niger"[MeSH Terms] OR "niger"[All Fields]) OR ("nigeria"[MeSH Terms] OR "nigeria"[All Fields] OR "nigeria s"[All Fields]) OR (("sierra"[All Fields] OR "sierras"[All Fields]) AND "Eone"[All Fields]) OR ("sierra leone"[MeSH Terms] OR ("sierra"[All Fields] AND "leone"[All Fields]) OR "sierra leone"[All Fields]) OR ("togo"[MeSH Terms] OR "togo"[All Fields])

"fascioliasis"[MeSH Terms] OR "fascioliasis"[All Fields] OR ("fasciola hepatica"[MeSH Terms] OR ("fasciola"[All Fields] AND "hepatica"[All Fields]) OR "fasciola hepatica"[All Fields] OR ("liver"[All Fields] AND "fluke"[All Fields]) OR "liver fluke"[All Fields]) OR (("fasciola"[MeSH Terms] OR "fasciola"[All Fields]) AND "gigantica"[All Fields]) OR ("fasciola hepatica"[MeSH Terms] OR ("fasciola"[All Fields] AND "hepatica"[All Fields]) OR "fasciola hepatica"[All Fields]) OR ("fasciola"[MeSH Terms] OR "fasciola"[All Fields] OR ("fasciola"[All Fields] AND "sp"[All Fields]) OR "fasciola sp"[All Fields])

"infect"[All Fields] OR "infectability"[All Fields] OR "infectable"[All Fields] OR "infectant"[All Fields] OR "infectants"[All Fields] OR "infected"[All Fields] OR "infecteds"[All Fields] OR "infectibility"[All Fields] OR "infectible"[All Fields] OR "infecting"[All Fields] OR "infection s"[All Fields] OR

"infections"[MeSH Terms] OR "infections"[All Fields] OR "infection"[All Fields] OR "infective"[All Fields] OR "infectiveness"[All Fields] OR "infectives"[All Fields] OR "infectivities"[All Fields] OR "infects"[All Fields] OR "pathogenicity"[MeSH Subheading] OR "pathogenicity"[All Fields] OR "infectivity"[All Fields] OR (("infect"[All Fields] OR "infectability"[All Fields] OR "infectable"[All Fields] OR "infectant"[All Fields] OR "infectants"[All Fields] OR "infected"[All Fields] OR "infecteds"[All Fields] OR "infectibility"[All Fields] OR "infectible"[All Fields] OR "infecting"[All Fields] OR "infection s"[All Fields] OR "infections"[MeSH Terms] OR "infections"[All Fields] OR "infection"[All Fields] OR "infective"[All Fields] OR "infectiveness"[All Fields] OR "infectives"[All Fields] OR "infectivities"[All Fields] OR "infects"[All Fields] OR "pathogenicity"[MeSH Subheading] OR "pathogenicity"[All Fields] OR "infectivity"[All Fields]) AND ("j rehabil assist technol eng"[Journal] OR "rate"[All Fields])) OR ("intense"[All Fields] OR "intensely"[All Fields] OR "intensities"[All Fields] OR "intensity"[All Fields] OR "intensively"[All Fields]) OR ("epidemiology"[MeSH Subheading] OR "epidemiology"[All Fields] OR "prevalence"[All Fields] OR "prevalence"[MeSH Terms] OR "prevalance"[All Fields] OR "prevalences"[All Fields] OR "prevalence s"[All Fields] OR "prevalent"[All Fields] OR "prevalently"[All Fields] OR "prevalents"[All Fields]) OR ("epidemiology"[MeSH Subheading] OR "epidemiology"[All Fields] OR "incidence"[All Fields] OR "incidence"[MeSH Terms] OR "incidences"[All Fields] OR "incident"[All Fields] OR "incidents"[All Fields])

(("lymnaea"[MeSH Terms] OR "lymnaea"[All Fields] OR "lymnaeas"[All Fields]) AND ("columella"[All Fields] OR "columellae"[All Fields] OR "columellas"[All Fields])) OR (("lymnaea"[MeSH Terms] OR "lymnaea"[All Fields] OR "lymnaeas"[All Fields]) AND "natalensis"[All Fields]) OR (("lymnaea"[MeSH Terms] OR "lymnaea"[All Fields] OR "lymnaeas"[All Fields]) AND "truncatula"[All Fields]) OR ("Pseudosuccinea"[All Fields] AND ("columella"[All Fields] OR "columellae"[All Fields] OR "columellas"[All Fields])) OR (("plant roots"[MeSH Terms] OR ("plant"[All Fields] AND "roots"[All Fields]) OR "plant roots"[All Fields] OR "radix"[All Fields] OR "radixes"[All Fields]) AND "natalensis"[All Fields]) OR ("Galba"[All Fields] AND "truncatula"[All Fields])

(("fasciola"[MeSH Terms] OR "fasciola"[All Fields]) AND ("intermediate"[All Fields] OR "intermediated"[All Fields] OR "intermediately"[All Fields] OR "intermediates"[All Fields]) AND "host"[All Fields]) OR (("intermediate"[All Fields] OR "intermediated"[All Fields] OR "intermediately"[All Fields] OR "intermediates"[All Fields]) AND "host"[All Fields] AND ("snail s"[All Fields] OR "snails"[MeSH Terms] OR "snails"[All Fields] OR "snail"[All Fields])) OR (("fresh water"[MeSH Terms] OR ("fresh"[All Fields] AND "water"[All Fields]) OR "fresh water"[All Fields] OR "freshwater"[All Fields] OR "freshwaters"[All Fields]) AND ("snail s"[All Fields] OR "snails"[MeSH Terms] OR "snails"[All Fields] OR "snail"[All Fields])) OR (("snail s"[All Fields] OR "snails"[MeSH Terms] OR "snails"[All Fields] OR "snail"[All Fields]) AND ("genetic vectors"[MeSH Terms] OR ("genetic"[All Fields] AND "vectors"[All Fields]) OR "genetic vectors"[All Fields] OR "vector"[All Fields] OR "vectors"[All Fields] OR "vector s"[All Fields] OR "vectored"[All Fields] OR "vectoring"[All Fields] OR "vectorization"[All Fields] OR "vectorize"[All Fields] OR "vectorized"[All Fields] OR "vectorizing"[All Fields])) OR ("Malacology"[All Fields] AND ("survey s"[All Fields] OR "surveyed"[All Fields] OR "surveying"[All Fields] OR "surveys and questionnaires"[MeSH Terms] OR ("surveys"[All Fields] AND "questionnaires"[All Fields]) OR "surveys and questionnaires"[All Fields] OR "survey"[All Fields] OR "surveys"[All Fields])) "togo"[MeSH Terms] OR "togo"[All Fields] "sierra leone"[MeSH Terms] OR ("sierra"[All Fields] AND "leone"[All Fields]) OR "sierra leone"[All Fields] ("sierra"[All Fields] OR "sierras"[All Fields]) AND "Eone"[All Fields] "nigeria"[MeSH Terms] OR "nigeria"[All Fields] OR "nigeria s"[All Fields] "niger"[MeSH Terms] OR "niger"[All Fields] "mauritania"[MeSH Terms] OR "mauritania"[All Fields] "mali"[MeSH Terms] OR "mali"[All Fields] "liberia"[MeSH Terms] OR "liberia"[All Fields] OR "liberia s"[All Fields] "guinea bissau"[MeSH Terms] OR "guinea bissau"[All Fields] OR ("guinea"[All Fields] AND "bissau"[All Fields]) OR "guinea bissau"[All Fields]

"guinea"[MeSH Terms] OR "guinea"[All Fields] OR "guinea s"[All Fields] OR "guineas"[All Fields] "ghana"[MeSH Terms] OR "ghana"[All Fields] OR "ghana s"[All Fields] "gambia"[MeSH Terms] OR "gambia"[All Fields] OR "gambia s"[All Fields] "cote d ivoire"[MeSH Terms] OR ("cote"[All Fields] AND "d ivoire"[All Fields]) OR "cote d ivoire"[All Fields]"cabo verde"[MeSH Terms] OR ("cabo"[All Fields] AND "verde"[All Fields]) OR "cabo verde"[All Fields] OR ("cape"[All Fields] AND "verde"[All Fields]) OR "cape verde"[All Fields] "burkina faso"[MeSH Terms] OR ("burkina"[All Fields] AND "faso"[All Fields]) OR "burkina faso"[All Fields]"benin"[MeSH Terms] OR "benin"[All Fields] OR "benin s"[All Fields]"uganda"[MeSH Terms] OR "uganda"[All Fields] OR "uganda s"[All Fields] "tanzania"[MeSH Terms] OR "tanzania"[All Fields] OR "tanzania s"[All Fields] "somalia"[MeSH Terms] OR "somalia"[All Fields] OR "somalia s"[All Fields] "seychelles"[MeSH Terms] OR "seychelles"[All Fields] "rwanda"[MeSH Terms] OR "rwanda"[All Fields] OR "rwanda s"[All Fields] "kenya"[MeSH Terms] OR "kenya"[All Fields] OR "kenya s"[All Fields] "eritrea"[MeSH Terms] OR "eritrea"[All Fields] "ethiopia"[MeSH Terms] OR "ethiopia"[All Fields] OR "ethiopia s"[All Fields] "djibouti"[MeSH Terms] OR "djibouti"[All Fields] "comoros"[MeSH Terms] OR "comoros"[All Fields] OR "comoro"[All Fields] "burundi"[MeSH Terms] OR "burundi"[All Fields] "mauritius"[MeSH Terms] OR "mauritius"[All Fields] "malawi"[MeSH Terms] OR "malawi"[All Fields] OR "malawi s"[All Fields] "madagascar"[MeSH Terms] OR "madagascar"[All Fields] OR "madagascar s"[All Fields] "eswatini"[MeSH Terms] OR "eswatini"[All Fields] OR "swaziland"[All Fields] "mauritius"[MeSH Terms] OR "mauritius"[All Fields] "mozambique"[MeSH Terms] OR "mozambique"[All Fields] OR "mozambique s"[All Fields] "mozambique"[MeSH Terms] OR "mozambique"[All Fields] OR "mozambique s"[All Fields] "zambia"[MeSH Terms] OR "zambia"[All Fields] OR "zambia s"[All Fields] "zimbabwe"[MeSH Terms] OR "zimbabwe"[All Fields] OR "zimbabwe s"[All Fields] "south africa"[MeSH Terms] OR ("south"[All Fields] AND "africa"[All Fields]) OR "south africa"[All Fields] "namibia"[MeSH Terms] OR "namibia"[All Fields] OR "namibia s"[All Fields] "lesotho"[MeSH Terms] OR "lesotho"[All Fields] "botswana"[MeSH Terms] OR "botswana"[All Fields] OR "botswana s"[All Fields] "Sao"[All Fields] AND "Tome"[All Fields] AND ("principe"[All Fields] OR "principes"[All Fields]) "Sao"[All Fields] AND "Tome"[All Fields] "gabon"[MeSH Terms] OR "gabon"[All Fields] "equatorial guinea" [MeSH Terms] OR ("equatorial"[All Fields] AND "guinea"[All Fields]) OR "equatorial guinea"[All Fields] ("democrat"[All Fields] OR "democratic"[All Fields] OR "democratically"[All Fields] OR "democratization"[All Fields] OR "democratize"[All Fields] OR "democratized"[All Fields] OR "democratizing"[All Fields] OR "democrats"[All Fields]) AND ("republic"[All Fields] OR "republic s"[All Fields] OR "republics"[All Fields]) AND ("congo"[MeSH Terms] OR "congo"[All Fields]) "Brazzaville"[All Fields]("congo"[MeSH Terms] OR "congo"[All Fields]) AND ("republic"[All Fields] OR "republic s"[All Fields] OR "republics"[All Fields])"chad"[MeSH Terms] OR "chad"[All Fields]"central african republic"[MeSH Terms] OR ("central"[All Fields] AND "african"[All Fields] AND "republic"[All Fields]) OR "central african republic"[All Fields]"cameroon"[MeSH Terms] OR "cameroon"[All Fields] OR "cameroons"[All Fields] OR "cameroon s"[All Fields] "angola"[MeSH Terms] OR "angola"[All Fields] OR "angola s"[All Fields] "tunisia"[MeSH Terms] OR "tunisia"[All Fields] "sudan"[MeSH Terms] OR "sudan"[All Fields] OR "sudans"[All Fields] OR "sudan s"[All Fields]"Morroco"[All Fields]"libya"[MeSH Terms] OR "libya"[All Fields]"egypt"[MeSH Terms] OR "egypt"[All Fields] OR "egypt s"[All Fields]"algeria"[MeSH Terms] OR "algeria"[All Fields]

"africa"[MeSH Terms] OR "africa"[All Fields] OR "africa s"[All Fields] OR "africas"[All Fields]

"fasciola"[MeSH Terms] OR "fasciola"[All Fields] OR ("fasciola"[All Fields] AND "sp"[All Fields]) OR "fasciola sp"[All Fields]

"fasciola hepatica"[MeSH Terms] OR ("fasciola"[All Fields] AND "hepatica"[All Fields]) OR "fasciola hepatica"[All Fields]

("fasciola"[MeSH Terms] OR "fasciola"[All Fields]) AND "gigantica"[All Fields]

"fasciola hepatica"[MeSH Terms] OR ("fasciola"[All Fields] AND "hepatica"[All Fields]) OR "fasciola hepatica"[All Fields] OR ("liver"[All Fields] AND "fluke"[All Fields]) OR "liver fluke"[All Fields]"fascioliasis"[MeSH Terms] OR "fascioliasis"[All Fields]

"epidemiology"[MeSH Subheading] OR "epidemiology"[All Fields] OR "incidence"[All Fields] OR "incidence"[MeSH Terms] OR "incidences"[All Fields] OR "incident"[All Fields] OR "incidents"[All Fields]

"epidemiology"[MeSH Subheading] OR "epidemiology"[All Fields] OR "prevalence"[All Fields] OR "prevalence"[MeSH Terms] OR "prevalance"[All Fields] OR "prevalences"[All Fields] OR "prevalence s"[All Fields] OR "prevalent"[All Fields] OR "prevalently"[All Fields] OR "prevalents"[All Fields]

"intense"[All Fields] OR "intensely"[All Fields] OR "intensities"[All Fields] OR "intensity"[All Fields] OR "intensively"[All Fields]

("infect"[All Fields] OR "infectability"[All Fields] OR "infectable"[All Fields] OR "infectant"[All Fields] OR "infectants"[All Fields] OR "infected"[All Fields] OR "infecteds"[All Fields] OR "infectibility"[All Fields] OR "infectible"[All Fields] OR "infecting"[All Fields] OR "infection s"[All Fields] OR "infections"[MeSH Terms] OR "infections"[All Fields] OR "infection"[All Fields] OR "infective"[All Fields] OR "infectiveness"[All Fields] OR "infectives"[All Fields] OR "infectivities"[All Fields] OR "infects"[All Fields] OR "pathogenicity"[MeSH Subheading] OR "pathogenicity"[All Fields] OR "infectivity"[All Fields]) AND ("j rehabil assist technol eng"[Journal] OR "rate"[All Fields])

"infect"[All Fields] OR "infectability"[All Fields] OR "infectable"[All Fields] OR "infectant"[All Fields] OR "infectants"[All Fields] OR "infected"[All Fields] OR "infecteds"[All Fields] OR "infectibility"[All Fields] OR "infectible"[All Fields] OR "infecting"[All Fields] OR "infection s"[All Fields] OR "infections"[MeSH Terms] OR "infections"[All Fields] OR "infection"[All Fields] OR "infective"[All Fields] OR "infectiveness"[All Fields] OR "infectives"[All Fields] OR "infectivities"[All Fields] OR "infects"[All Fields] OR "pathogenicity"[MeSH Subheading] OR "pathogenicity"[All Fields] OR "infectivity"[All Fields]

"Galba"[All Fields] AND "truncatula"[All Fields]

("plant roots"[MeSH Terms] OR ("plant"[All Fields] AND "roots"[All Fields]) OR "plant roots"[All Fields] OR "radix"[All Fields] OR "radixes"[All Fields]) AND "natalensis"[All Fields]

"Pseudosuccinea"[All Fields] AND ("columella"[All Fields] OR "columellae"[All Fields] OR "columellas"[All Fields])

("lymnaea"[MeSH Terms] OR "lymnaea"[All Fields] OR "lymnaeas"[All Fields]) AND "truncatula"[All Fields]

("lymnaea"[MeSH Terms] OR "lymnaea"[All Fields] OR "lymnaeas"[All Fields]) AND "natalensis"[All Fields]

("lymnaea"[MeSH Terms] OR "lymnaea"[All Fields] OR "lymnaeas"[All Fields]) AND ("columella"[All Fields] OR "columellae"[All Fields] OR "columellas"[All Fields])

"Malacology"[All Fields] AND ("survey s"[All Fields] OR "surveyed"[All Fields] OR "surveying"[All Fields] OR "surveys and questionnaires"[MeSH Terms] OR ("surveys"[All Fields] AND "questionnaires"[All Fields]) OR "surveys and questionnaires"[All Fields] OR "survey"[All Fields] OR "surveys"[All Fields])

("snail s"[All Fields] OR "snails"[MeSH Terms] OR "snails"[All Fields] OR "snail"[All Fields]) AND ("genetic vectors"[MeSH Terms] OR ("genetic"[All Fields] AND "vectors"[All Fields]) OR "genetic vectors"[All Fields] OR "vector"[All Fields] OR "vectors"[All Fields] OR "vector s"[All Fields] OR "vectored"[All Fields] OR "vectoring"[All Fields] OR "vectorization"[All Fields] OR "vectorize"[All Fields] OR "vectorized"[All Fields] OR "vectorizing"[All Fields])

("fresh water"[MeSH Terms] OR ("fresh"[All Fields] AND "water"[All Fields]) OR "fresh water"[All Fields] OR "freshwater"[All Fields] OR "freshwaters"[All Fields]) AND ("snail s"[All Fields] OR "snails"[MeSH Terms] OR "snails"[All Fields] OR "snail"[All Fields])

("intermediate"[All Fields] OR "intermediated"[All Fields] OR "intermediately"[All Fields] OR "intermediates"[All Fields]) AND "host"[All Fields] AND ("snail s"[All Fields] OR "snails"[MeSH Terms] OR "snails"[All Fields] OR "snail"[All Fields])

("fasciola"[MeSH Terms] OR "fasciola"[All Fields]) AND ("intermediate"[All Fields] OR "intermediated"[All Fields] OR "intermediately"[All Fields] OR "intermediates"[All Fields]) AND "host"[All Fields]
