# Supplementary material for: Infection Rates of Fasciola Intermediate Host Snail Species and Their Distribution in Africa: A Systematic Review and Meta-Analysis
Source: Trop Med Infect Dis. 2023 Oct 6;8(10):467. doi: 10.3390/tropicalmed8100467 (PMC10610779; doi:10.3390/tropicalmed8100467)
Supplement: Supplementary file 1 [file tropicalmed-08-00467-s001.zip › File S2 Quality assessment.pdf]

Quality assessment of the articles included.

The Joanna Briggs Institute Critical Appraisal Tool for studies reporting prevalence data was used to assess the quality of all studies included in the review. The following questions were accessed using the tool assessed the about the articles:

1. sample representative of the target population,
2. study participants recruited in an appropriate way,
3. sample size adequate
4. study subjects and setting described in detail
5. data analysis conducted with sufficient coverage of the identified sample
6. objective standard criteria used for measurement of the condition
7. condition measured reliably,
8. appropriate statistical analysis,
9. important confounding factors/ subgroups/differences identified and accounted for,
10. subpopulations identified using objective criteria.

First Author and Year	Was the sample representative of the target population?	Were study participants recruited in an appropriate way?	Was the sample size adequate?	Were the study subjects and setting described in detail?	Is the data analysis conducted with sufficient coverage of the identified sample?	Were objective, standard criteria used for measurement of the condition?	Was the condition measured reliably?	Was there appropriate statistical analysis?	Are all important confounding factors/ subgroups/differences identified and accounted for?	Were subpopulations identified using objective criteria?	Quality score
Ibrahim and Ahmed. (2019)	Yes	Yes	Yes	Yes	Yes	Yes	Yes	Yes	Yes	Yes	10
Ashour et al. (2008)	Yes	Yes	Yes	Yes	Yes	Yes	Yes	Yes	Yes	Yes	10
El Shazly et al. (2002)	Yes	Yes	Yes	Yes	Yes	Yes	Yes	Yes	No	Yes	9
Ahmed and Ramzy (1999)	Yes	Yes	Yes	Yes	Yes	Yes	Yes	Yes	Yes	Yes	10
Bushara and Majid. (2001)	Yes	Yes	Yes	Yes	Yes	Yes	Yes	Yes	No	Yes	9
Califa and Mas-Coma. (2005)	Yes	Yes	Yes	Yes	Yes	Yes	Yes	Yes	Yes	Yes	10
Kele and Attah. (2022)	Yes	Yes	Yes	Yes	Yes	Yes	Yes	Yes	Yes	Yes	10
Bagbe (2017)	Yes	Yes	Yes	Yes	Yes	Yes	Yes	Yes	Yes	Yes	10
Luka and Mbaya. (2015)	Yes	Yes	Yes	Yes	Yes	Yes	Yes	Yes	Yes	Yes	10
Mailkaje and Umar. (2018)	Yes	Yes	Yes	Yes	Yes	Yes	Yes	Yes	Yes	Yes	10
Mereta et al. (2019)	Yes	Yes	Yes	Yes	Yes	Yes	Yes	Yes	Yes	Yes	10
Phiri et al. (2007)	Yes	Yes	Yes	Yes	Yes	Yes	Yes	Yes	Yes	Yes	10
Nkwengulila and Kigadye. (2005)	Yes	Yes	Yes	Yes	Yes	Yes	Yes	Yes	Yes	Yes	10
Deribew et al. (2020)	Yes	Yes	Yes	Yes	Yes	Yes	Yes	Yes	Yes	Yes	10
Righi, et al. (2016)	Yes	Yes	Yes	Yes	Yes	Yes	Yes	Yes	No	Yes	9
Hammami, et al. (2007)	Yes	Yes	Yes	Yes	Yes	Yes	Yes	Yes	Yes	Yes	10
Arafa et al. (2018)	Yes	Yes	Yes	Yes	Yes	Yes	Yes	Yes	Yes	Yes	10
El Shazly, et al. (2002)	Yes	Yes	Yes	Yes	Yes	Yes	Yes	Yes	No	Yes	9
Mekroud, et al. (2004)	Yes	Yes	Yes	Yes	Yes	Yes	Yes	Yes	Yes	Yes	10
Malatji and Mukaratirwa (2020)	Yes	Yes	Yes	Yes	Yes	Yes	Yes	Yes	Yes	Yes	10
Ahmed and Ramzy (1999)	Yes	Yes	Yes	Yes	Yes	Yes	Yes	Yes	Yes	Yes	10
Grabner et al. (2014)	Yes	Yes	Yes	Yes	Yes	Yes	Yes	Yes	No	Yes	9
El Shazly et al. (2002)	Yes	Yes	Yes	Yes	Yes	Yes	Yes	Yes	No	Yes	9
